# Nutrient use and methane emissions in growing beef fed different protein sources and a pasture-based diet

**DOI:** 10.1093/jas/skaf007

**Published:** 2025-01-17

**Authors:** Christos Christodoulou, Kirsty E Kliem, Marc D Auffret, David J Humphries, Paul Kirton, Hassan Jalal, John R Newbold, Nicholas Davison, Laurence G Smith, Sokratis Stergiadis

**Affiliations:** School of Agriculture, Policy and Development, University of Reading, Reading RG6 6EU, UK; School of Agriculture, Policy and Development, University of Reading, Reading RG6 6EU, UK; Agrifirm, B-9031 Gent (Drongen), Belgium; Centre for Dairy Research, School of Agriculture, Policy and Development, University of Reading, Reading RG2 9HX, UK; Centre for Dairy Research, School of Agriculture, Policy and Development, University of Reading, Reading RG2 9HX, UK; Department of Veterinary Medicine, University of Teramo, 64100 Teramo, Italy; Dairy Research and Innovation Centre, Scotland’s Rural College, Dumfries DG1 3NE, UK; School of Agriculture, Policy and Development, University of Reading, Reading RG6 6EU, UK; School of Agriculture, Policy and Development, University of Reading, Reading RG6 6EU, UK; Department of Biosystem and Teknologi, Swedish University of Agricultural Sciences, SE-234 22 Lomma, Sweden; School of Agriculture, Policy and Development, University of Reading, Reading RG6 6EU, UK

**Keywords:** alternative protein sources, beef production, energy utilization, greenhouse gas emissions, nutrient utilization

## Abstract

This study investigated the effects of different protein sources on feed intake, nutrient, and energy utilization, growth performance, and enteric methane (**CH**_**4**_) emissions in growing beef cattle, also evaluated against a pasture-based diet. Thirty-two Holstein × Angus growing beef were allocated to four dietary treatments: a total mixed ration (**TMR**) including solvent-extracted soybean meal as the main protein source (*n* = 8), TMR with local brewers’ spent grains (*n* = 8), TMR with local field beans (*n* = 8), and a diet consisting solely of fresh-cut Italian ryegrass (**GRA**; *n* = 8). Every 4 wk, animals were moved to digestibility stalls within respiration chambers to measure nutrient intakes, energy and nitrogen (**N**) utilization, and enteric CH_4_ emissions. Feed intake (Calan gates), nutrient intakes, and CH_4_ emissions (GreenFeed) were also measured when animals were group-housed. In respiratory chambers, enteric CH_4_ yield per kg of dry matter intake (**DMI**), per kg of organic matter intake (**OMI**), and per kg body weight were lower (*P* < 0.05) for GRA. Feces and urine energy outputs were higher (*P* = 0.007 and *P *< 0.001, respectively) for GRA steers than concentrate-fed steers. Urinary nitrogen output (**UNO**, *P *= 0.026), manure (feces + urine) nitrogen output (**MNO**, *P *= 0.034), UNO/nitrogen intake (*P *= 0.002), and MNO/nitrogen intake (*P *= 0.006) were higher for GRA. During group-housing periods, CH_4_ emissions, measured by GreenFeed, were similar to those measured in chambers. Similar CH_4_ yield between treatments, expressed per kg digestible DMI and digestible OMI, may indicate that the lower diet digestibility was likely the reason for the reduced enteric CH_4_ emissions in pasture-based diets. The higher energy output and nitrogen losses, and the reduced nitrogen utilization for steers fed the fresh-cut ryegrass diet indicate less efficient energy and nitrogen utilization, which can be considered environmentally undesirable. The lower growth rates in the pasture-based system should also be accounted for when this is adopted for reducing production costs.

## Teaser

This study aimed to evaluate the effects of local brewers’ spent grains and local field beans as alternative protein sources to soy. It also compares these concentrate-based diets with a pasture-based diet in terms of growth rates, nutrient balance, and enteric methane emissions. While pasture-based steers had lower enteric methane emissions per kilogram of dry matter and organic matter intake, their reduced energy and nitrogen use efficiency, along with the slower growth, may pose environmental challenges (e.g., more methane per unit of meat) and require careful consideration in terms of production costs.

## Graphical Abstract

**Graphical Abstract:**
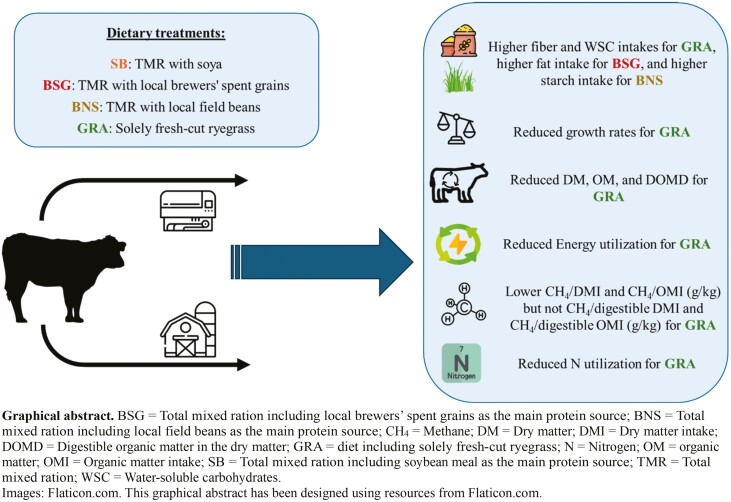

## Introduction

Evaluating and selecting protein sources in livestock diets is crucial for optimizing livestock performance, nutrient utilization, and environmental sustainability, particularly by reducing the use of protein sources with a high carbon footprint (Pexas et al., 2023). Protein sources such as soybean (*Glycine max*) meal are widely used in beef diets due to their high-protein content and favorable amino acid profile (Keller et al., 2021). However, the reliance on soy in livestock diets has raised environmental concerns, primarily due to its association with deforestation, excessive water use (Ferreira et al., 2016; Song et al., 2021), and financial challenges (market price volatility, etc.) (de Visser et al., 2014). Locally available protein sources may not carry a high carbon footprint associated with land use change and can reduce economic risks, and environmental footprint associated with feed transportation (Wägeli et al., 2015; Pexas et al., 2023).

Brewers’ spent grains, a coproduct of the brewing industry, could be an alternative to soybeans in ruminant rations, contribute to waste reduction in the food system, and promote a circular agricultural economy (Mussatto et al., 2006). Brewers’ spent grains are rich in fiber and relatively high in protein (21% dry matter; **DM**) (Zeko-Pivac et al., 2022). When replacing cracked wheat (*Triticum spp.*) grain and solvent-extracted canola (*Brassica napus L.*) meal with brewers’ grains in lactating dairy cow diets, a 5.2% lower methane (**CH**_**4**_) yield (g/kg DM intake; **DMI**) and a 9.1% reduction in CH_4_ intensity (g/L milk) was observed (Moate et al., 2011). Additionally, replacing grass silage with brewers’ grains in a barley-straw (*Hordeum vulgare L.*)-based diet for nonlactating cows during gestation resulted in up to 22.8% reduction in CH_4_ yield (g/kg DMI) (Duthie et al., 2015). These changes were potentially due to the higher dietary fat concentration when diets contained brewers’ grains. Despite this, it has not yet been evaluated as the main protein source for growing beef cattle and against soybean meal.

Field beans (*Vicia faba*) are another potential protein source that could replace soy in beef rations (Johnston et al., 2019). The use of local beans can enhance the sustainability of beef production systems by supporting local agricultural systems and reducing reliance on imported feed ingredients (Wägeli et al., 2015; Pexas et al., 2023). From a nutritional perspective, protein concentration is lower in field beans than in solvent-extracted soybean meal (280 vs. 470 g/kg DM), but the high starch content has additional nutritional value in ruminant diets (Johnston et al., 2019). However, both field beans and soybeans contain antinutritional factors such as trypsin inhibitors and tannins, which may have a negative effect on intake (Dvořák et al., 2006). As an alternative protein source for dairy cows, a dietary inclusion of up to 4.7 kg per cow per day was deemed acceptable, despite minor negative effects, such as a trend toward lower nitrogen use efficiency [milk N/N intake (**NI**)] ratios, reduced liver weights, and elevated blood urea nitrogen levels (Johnston et al., 2019).

To the best of our knowledge, this study is the first to evaluate UK-locally sourced brewers’ spent grains and field beans as alternative protein sources to soybean meal in growing beef rations and assess their effects on nutrient and energy utilization and enteric CH_4_ emissions. By evaluating these parameters, this study seeks to provide insights into the potential benefits of each protein source, thereby informing more sustainable and efficient beef-feeding practices.

The production of beef from pasture-based systems, without the supplementation of concentrate feeds, can reduce production costs (Pinheiro Machado Filho et al., 2021), support livelihoods and economies (Boval and Dixon, 2012), preserve and enhance biodiversity (Boval and Dixon, 2012; Fraser et al., 2022), and align with modern consumer demands (Klopatek et al., 2022). These systems are often preferred by consumers for their perceived benefits to animal health and welfare (Klopatek et al., 2022) and their more favorable nutritional profile, such as meat with more unsaturated and less saturated fatty acid (Średnicka-Tober et al., 2016; Ribas-Agustí et al., 2019; Clinquart et al., 2022; Klopatek et al., 2022).

Accordingly, the diets with different concentrate protein sources were compared against a pasture-based diet to assess the relative impact of different feeding strategies (concentrate-based vs. pasture) on animal performance and enteric CH_4_ emissions.

## Materials and Methods

### Animal ethics

All animal procedures were conducted following the UK Animals (Scientific Procedures) Act, 1986, following approval by the local animal welfare and ethical review board (DAS/C221Relivestock01).

### Experimental design and diets

Thirty-two Aberdeen Angus × Holstein cattle (16 steers, 16 heifers), born between June and August 2022 and raised in the Center for Dairy Research at the University of Reading, were used in a completely randomized continuous blocked design. Cattle were transferred to the Meat and Growth Research Unit at 12 wk of age. Animals were blocked and then randomly allocated within the block into four experimental groups of eight animals each (*n* = 8; 4 steers and 4 heifers per group) balanced for age, date of birth, and body weight (**BW**). The experiment started in mid-June of 2023 when animals were 332 ± 32 days of age and at a BW of 394 ± 30 kg. Animals from all experimental groups were housed together but individually fed in feeders with an electronic recognition system (Calan Broadbent Feeding System; Calan Gates, American Calan; NH, USA). Animals in the three concentrate-fed experimental groups were fed for 19 wk, total mixed rations (**TMRs**) based on maize and grass silages, at a forage:concentrate ratio of 64:36, with a supplementary blend of concentrate ingredients, either: (i) soy-based concentrate (**SB**), (ii) local brewers’ spent grains, comprised of the nonstarch parts of barley grains-based concentrate (**BSG**), and (iii) local field beans-based concentrate (**BNS**) (Table 1). Diets were formulated to be isonitrogenous and isoenergetic. The fourth experimental group was fed indoors exclusively fresh-cut Italian ryegrass (*Lolium multiflorum*, **GRA**; no concentrate) (Table 1). All animals across all groups also consumed some concentrate feed used as bait to encourage animals to access the automated head chamber systems (Greenfeed, C-Lock Inc.) for CH_4_ emissions measurements. Intake of this bait feed eventually constituted 7.9–8.7% of total DM across the different diets.

**Table 1.** Ingredient composition of experimental diets

Item	Dietary treatments^1^			
	SB	BSG	BNS	GRA
Forage:Concentrate ratio	64:36	64:36	64:36	91:9
**Diet ingredients (g/kg DM** ^2^)				
Fresh-cut Italian ryegrass	-	-	-	ad libitum
Grass silage	184	183	184	-
Corn silage	461	458	460	-
Soy-Hipro^3^	74	-	-	-
Soybean hulls	46	-	28	-
Sugar beet pulp unmolassed	64	-	-	-
Rapeseed meal^4^	46	46	46	-
Ground barley grain	37	37	37	-
Brewers’ spent grains	-	183	-	-
Ground field beans	-	-	156	-
Urea	-	2	-	-
Calf Starter pellet^5^	79	82	80	87
Trace elements and minerals (g/day)^6^	9	9	9	-

^1^SB = TMR including soy as the main protein source; BSG = TMR including local brewers’ spent grains as the main protein source; BNS = TMR including local field beans as the main protein source; GRA = diet including solely fresh-cut ryegrass.

^2^DM.

^3^Coproduct of oil manufacture obtained from dehulled soybeans after solvent extraction and subsequent heat treatment.

^4^Solvent-extracted rapeseed meal.

^5^Calf starter pellet as the GreenFeed bait consisting of wheat grain, rye grain, rapeseed meal, sunflower seed meal, palm kernel expeller meal, soybean hulls, sugar cane molasses, distillers’ dark maize grains, beans, calcium carbonate, sodium chloride, barley products, a natural antioxidant complex (10 iu vitamin E), vitamin A 8,000 iu, Vitamin D3 2,500 iu, and trace elements.

^6^Supplement containing: Vitamin A = 400,000 iu/kg, Vitamin D3 = 80,000 iu/kg, Vitamin E = 2,000 iu/kg, Vitamin B12 = 1,400 iu/kg, Calcium = 20%, Phosphorous = 5%, Magnesium = 5%, Salt = 20%.

### Grass growth management

Italian ryegrass was sown on September 13, 2022, at a 30-kg/ha seeding rate. The seedbed preparation involved cultivating the soil by plowing with a press, followed by power harrowing and drilling. The fertilization procedures for the field included the application of organic slurry manure at 42.615 m³/ha on February 2023, followed by 100-kg/ha mineral and 140-kg/ha granulated urea (46%) fertilization on April and May 2023 at 100 kg/ha and then again slurry manure on June and September at rates of 37.953 and 35.000 m^3^/ha, respectively. Growth was visually monitored through the winter (2022/2023) and once growth started in early spring. Available forage DM was estimated by a rising plate meter (FARMWORKS Precision Farming Systems, Feilding, New Zealand) by taking 20 sward height readings for each pre- and postcutting measurement. Sward DM yield estimations were calibrated every second day (between Monday and Friday) by taking 3 × 0.5 m^2^ quadrat cuts of the sward at a target postgrazing height of 7.0 cm and oven drying (100 °C) the sample to measure sward DM yield per m^2^ which was applied to each sward height measurement. To allow a cutting wedge to be formed, the first rotation was commenced when the ryegrass cover was between 2,100 and 2,400 kg DM/ha.

### Measurements in group-housed animals

Animals had ad libitum access to feed. Metabolizable energy requirements of the animals were assumed to be 10.5–11.5 MJ/kg DM and estimated DMI at 2.0–2.5% of BW; and a + 5% ME supply was provided according to the ME content of the experimental diets. Weekly feed intake was determined from daily measurements of feed offered and refusals. Moreover, BW was recorded weekly throughout the experiment on the same day, and at approximately the same time each day. The recorded BW was used to assess the BW change (**BWc**) and feed efficiency. Feed samples (TMRs and fresh-cut ryegrass) were collected weekly throughout the experiment, and an amount was oven-dried at 100 °C for DM determination, while a sub-sample was oven-dried at 65 °C and ground using a 1-mm screen for subsequent proximate analysis.

### GreenFeed

Enteric CH_4_ emissions were recorded daily from the third week onward, using an automated head chamber system (GreenFeed system; C-Lock, Inc., Rapid City, SD). Alleyway gates were installed in front of the GreenFeed unit, allowing only one animal to access a unit at any time. Each alleyway was designed to match the length of the animal, providing flank protection during measurements. A radio frequency identification reader identified each animal by its ear tag, and GreenFeed sampling was triggered once the animal’s head was positioned correctly within the unit’s hood, as detected by an infrared sensor. Animals could access the GreenFeed unit freely as long as it was unoccupied; however, access did not guarantee a CH_4_ measurement. Weekly calibrations with gas standards were conducted automatically by the GreenFeed units, and CO₂ recovery tests (100% ± 1.5) were performed monthly to ensure system accuracy. The airflow rates were above 27 L/s. The air filter was replaced weekly, and the used filter was cleaned and prepared for subsequent use. A ‘visit’ was recorded only when it resulted in a CH_4_ measurement. For a visit to be considered valid, a predefined time interval had to pass since the last recorded visit, and a food reward was dispensed to initiate a CH_4_ reading for that animal. Sampling also required a sufficient interval since the animal’s last CH_4_ measurement. The GreenFeed unit was programmed via C-Lock Inc. software to deliver ~35 g of pellets (as fed). The interval between bait supplement drops was set to 40 s, allowing for a maximum of six pellet drops per visit, resulting in a potential maximum visit duration of 240 s (4 min). The actual bait dispensed amounts and the number of drops were determined by the cup size and bait type used. Each animal was permitted a maximum of five visits per 24-h period, with a minimum interval of 3 h required between visits. Consequently, if an animal attempted to access the GreenFeed unit within 3 h of a previous visit, no pellets were dispensed. This setup allowed for precise control of feeding intervals and intake measurements, supporting reliable estimation of gas production per animal.

### Individual measurements in digestibility stalls and respiration chambers

Four steers within each group underwent additional digestibility assessments on four occasions until the end of the experiment. Every week, one animal from each dietary treatment underwent a 4-d (Monday to Friday) chamber measurement period of feed intake, total outputs of feces and urine, and respiration measurements, in individual digestibility stalls within individual respiration chambers. Individual DMI for each steer was calculated daily and averaged weekly. Bulked offered feeds and feed refusals were analyzed for DM by oven drying at 100 °C and a sub-sample was oven dried at 65 °C and then ground (1-mm screen) and stored for proximate analysis.

Feces collection chutes and urine hoppers were predesigned to ensure separation and no mixing of feces and urine during the 4-d collection period. Feces were collected into a tray via a bespoke chute, designed on-site, and emptied into a large bucket at regular intervals during the day and placed into a separate container. The chute was made from 100% phthalate-free PVC for the surface and 100% recycled polyester for the backing, ensuring durability during sample collection. Each morning feces were mixed and homogenized in a bucket and then stored at −20 °C. At the end of the 4-d collection period, the fecal samples from each day were thawed, thoroughly mixed, and homogenized, and 10% of the total feces weight was subsampled in a sealed container. The pooled samples were stored at −20 °C until analysis of N and gross energy (**GE**) contents. Further subsamples were aliquoted and analyzed for DM content (oven drying at 65 °C), and the dried samples were stored at room temperature before ash, neutral detergent fiber (**NDF**), and acid detergent fiber (**ADF**) content analysis. In addition, urine hoppers were predesigned for urine collection. Urine was collected under vacuum via a separator funnel strapped over the sheath into a 25-L container containing 1,200 mL of 10 N sulphuric acid to maintain urine pH < 2.0. The urine collection container was constantly stirred using a magnetic stirrer plate and flea to ensure the mixing of the acid and urine. The total daily urine collected was weighed and thoroughly mixed, and 10% of the total urine weight subsample was frozen at −20 °C in a sealed plastic bottle. After the 4-d collection period, the urine subsamples were thawed, pooled, and stored at −20 °C for later N content and GE analysis.

Respiration chamber assessments included (i) BW at the beginning and the end of the 4-d measurement phase, (ii) DM and proximate analysis of fresh-cut ryegrass and TMRs by wet-chemistry (N, GE, NDF, ADF, oil, ether extract (**EE**), starch, water-soluble carbohydrates (**WSC**), and ash, Table 2), (iii) feed intake and total outputs of feces and urine, (iv) feces (N, GE, EE, ADF, NDF, ash) and urine (N, GE) proximate analysis, and (v) gaseous exchange (O_2_, CO_2,_ and CH_4_) in respiration chambers. These records were used to measure: (i) feed use efficiency, (ii) nutrient digestibility (DM, organic matter; OM, NDF, and ADF), (iii) N use efficiency, and more specifically NI and N outputs in feces (**FNO**), urine (**UNO**), and manure (feces plus urine; **MNO**), as well as retained N as a proportion of NI, (iv) energy use efficiency (intake and output, digestible energy [**DE**], metabolizable energy [**ME**], DE/GE, ME/GE, ME/DE), and (iv) CH_4_ emissions (g/d and g/kg BW) and yield (g/kg of intakes of DM, OM, GE, DE and ME, digestible DM, and digestible OM).

**Table 2.** Average chemical composition and DM and gross energy concentration of the experimental diets during the 16 wk of the animal trial

Item	Dietary treatments^1^			
	SB (*n* = 16)	BSG (*n* = 16)	BNS (*n* = 16)	GRA (*n* = 16)
Dry matter (DM; g/kg fresh)	395	331	394	187
Organic matter (g/kg DM)	947	951	951	901
Gross energy (MJ/kg DM)	16.9	18.0	17.0	18.1
**Nutrients (g/kg DM)**				
Crude protein	124	128	116	135
Neutral detergent fiber	420	464	395	599
Acid detergent fiber	261	269	236	382
Oil	32.0	49.6	30.6	29.3
Ether extract	23.6	40.0	22.8	19.9
Starch	207	197	259	-
Water soluble carbohydrates	28.8	18.0	24.8	73.4
Ash	52.7	49.3	49.2	98.8

^1^SB = TMR including soy as the main protein source; BSG = TMR including local brewers’ spent grains as the main protein source; BNS = TMR including local field beans as the main protein source; GRA = diet including solely fresh-cut ryegrass.

### Sample analysis

Fresh samples of TMRs, fresh-cut ryegrass, and feces were oven-dried at 65 °C and milled (1-mm screen). Freeze-dried samples of TMRs, fresh-cut ryegrass, feces, and urine were analyzed for GE with combustion using an adiabatic bomb calorimeter. Freeze-dried samples of TMRs, fresh-cut ryegrass, feces, and urine were analyzed for GE with combustion using an adiabatic bomb calorimeter. For GE analysis, urine was thoroughly mixed and weighed into 6.6-cm sections of precut lay-flat tubing. The urine samples also included the H_2_SO4 at a concentration of 10 N. One end of the tubing was carefully double-sealed using a bag sealer. The samples were then freeze-dried, folded, and placed in crucibles. Approximately, 20 s were allowed for the samples to settle in place before analysis. The procedure followed the manufacturer’s instructions for the calorimeter bomb (Parr Instrument Company, Moline, IL). Samples of TMRs, fresh-cut ryegrass, and feces were also oven-dried at 100 °C for DM (988.05) and for ash content by combustion at 600 °C (942.05) (AOAC, 2012). For N analysis, fresh and not dried sample of feeds and feces was used, thus TMRs, fresh-cut ryegrass, feces, and urine samples were analyzed for N using the Kjeldahl method (AOAC, 2012), and crude protein (**CP**) was determined as N × 6.25. Feeds and feces samples were also analyzed for NDF, and ADF based on Roberston and Van Soest (1981) and Mertens (2002). Dried and milled feed samples were also analyzed for starch and WSC content on a continuous flow autoanalyzer system according to previously described methods (Smith et al., 1964; Fuller, 1967; MacRae and Armstrong, 1968). Furthermore, in dried and milled feed samples, oil content was determined by the modified “Wiebul” acid hydrolysis method, and EE content by direct solvent extraction (Soxhlet, 1879).

### Data analysis

**Figure 1.**
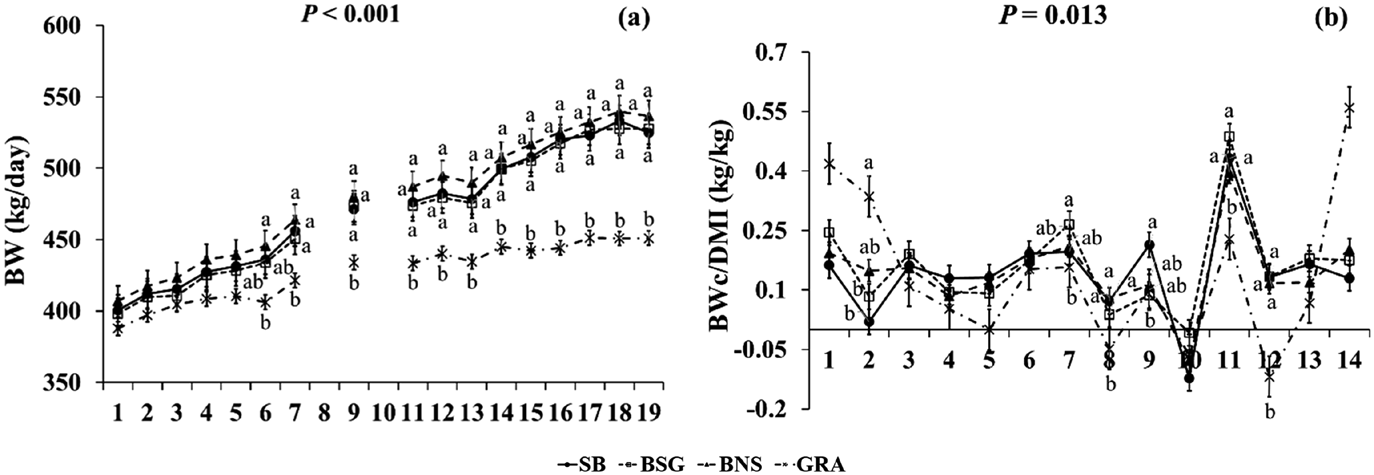
Significant effect of Treatment × Week interaction on BW (a) and BWc/DMI ratio (b), in growing beef (heifers, steers) fed the experimental diets during the group-housed period of the animal trial. Significances were declared at *P *< 0.05. Significant differences within weeks are indicated with different superscript letters according to Fisher’s LSD test (*P* < 0.05). Error bars represent standard error.

**Figure 2.**
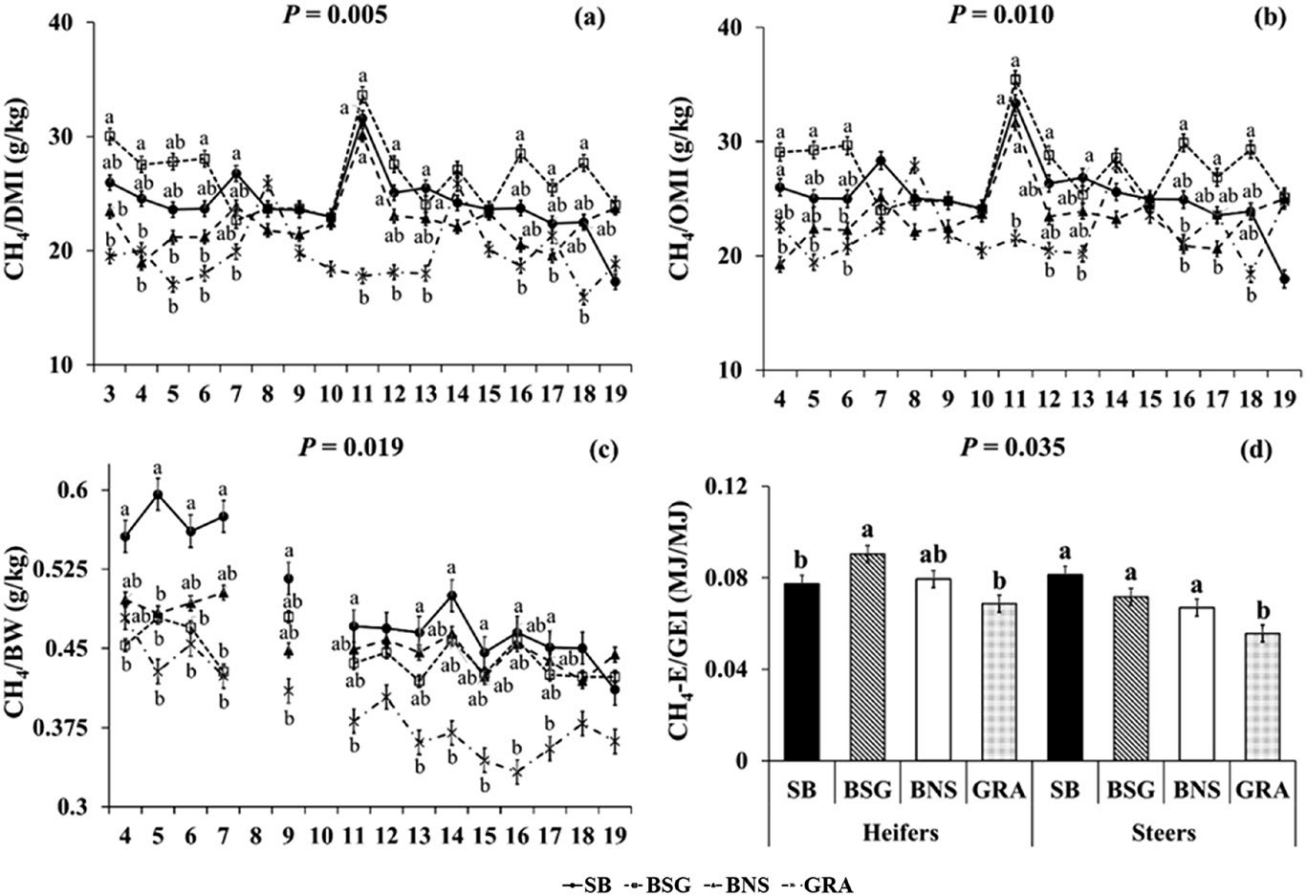
Significant effect of Treatment × Week interaction on (i) enteric CH_4_ emissions (expressed as g of CH_4_ per kg DMI, panel a; kg OMI, panel b; and kg BW, panel c), (ii) Treatment × Sex interaction on enteric CH_4_ emissions expressed as MJ per MJ GEI (panel d); in growing beef (heifers, steers) fed the experimental diets during the group-housed period of the animal trial. Significances were declared at *P *< 0.05. Significant differences within weeks (i) and sex (ii) are indicated with different superscript letters according to Fisher’s LSD test (*P* < 0.05). Error bars represent standard error.

Statistical analysis was conducted using IBM SPSS, version 29.0 (Armonk, NY, USA). Enteric CH_4_ emission data points represented average daily CH_4_ emissions (g/day) for individual animals across each chamber measurement period. The residuals of all variables were tested for normality, with the Kolmogorov–Smirnov test, and homogeneity with Lavene’s test; no variables showed deviation from normality and were all analyzed untransformed. Data were analyzed using a linear mixed effects model with dietary treatments (SB, BGS, BNS, GRA), the measurement week (repeated measurement), the dietary Treatment × Week for the group-housed period, and the dietary Treatment × Period for the respiration chamber measurement periods, and block being used as fixed factors, and animal (nested within dietary treatment) as a random factor. Repeated effects of the week within animals used covariance structure (compound symmetry, heterogeneous compound symmetry, autoregressive, heterogeneous autoregressive, diagonal, ante-dependence, or unstructured) giving the best fit based on the lowest Bayesian information criterion value for each variable of interest and based on the homogeneity of each variable. Where necessary, Fisher’s LSD Test (*P *< 0.05) was used for the means pairwise comparisons. Results are presented in Tables 1–6, Figures 1 and 2, and Supplementary Figures S1 and S2. The same statistical procedures were also used to compare the three concentrate-fed treatments (SB, BSG, BNS; by excluding the pasture-based treatment) and results are presented in Supplementary Tables S1–S4 and Supplementary Figures S3–S5.

## Results

### Feed and nutrient intakes, growth measurements, and enteric CH_4_ emissions during the group-housed period

For SB and BNS, DMI was higher (*P *= 0.002) compared with BSG and GRA and organic matter intake (**OMI**) was higher (*P *< 0.001) for the SB and BNS compared with GRA, while BSG had also lower (*P *< 0.001) compared with BNS (Table 3). There were no significant differences (*P *> 0.05) for GE intake and NI between the dietary treatments. Intakes of NDF and ADF were higher (*P *< 0.001) for GRA compared with the three concentrate-fed treatments, while ADF intake was also higher (*P *< 0.001) for SB compared with BSG and BNS. Both oil and EE intakes were higher (*P *< 0.001) for BSG compared with the other treatments and for SB and BNS compared with GRA. Starch intake was higher (*P *< 0.001) for BNS than SB and BSG, while WSC intake was higher (*P *< 0.001) for GRA than the rest, for SB than BSG and BNS, and for BNS than BSG. Beef of the three concentrate-fed treatments had also higher BW (*P *= 0.018, 39–51 kg) and BWc (*P *< 0.001, 0.64–0.86 kg/day) compared with those of GRA. The BWc/DMI ratio did not (*P *> 0.05) differ between the treatments. When the three concentrate-fed experimental treatments were compared after the GRA data were excluded from the dataset (as shown in Supplementary Material), DMI and OMI were significantly higher (*P *= 0.007 and *P *= 0.002, respectively) for SB and BNS than BSG. Oil and EE intakes were higher (*P *< 0.001) for BSG than SB and BNS, while starch intake was higher (*P *< 0.001) for BNS than SB and BSG and for SB than BSG. WSC intake was higher (*P *< 0.001) for SB than BSG and BNS and for BNS than BSG, while BW, BWc, and BWc/DMI did not differ (*P *> 0.05).

**Table 3.** Feed and nutrient intakes (kg/day), growth measurements, and enteric CH_4_ emissions from growing beef (heifers, steers) fed the experimental diets during the group-housed period of the animal trial

Item^3^	Dietary treatments (D)^1^					Sex (S)			*P*-values^2^						
	SB	BSG	BNS	GRA	SEM	Heifers	Steers	SEM	D	Week (W)	(S)	D × W	D × S	W × S	D × W × S
**Feed and nutrient intakes (kg/day)**															
DM^4^	9.78^a^	8.50^b^	9.74^a^	8.60^b^	0.276	8.41	9.89	0.195	0.002	<0.001	<0.001	0.007	0.926	0.396	1.000
OM^5^	9.44^ab^	8.29^bc^	9.58^a^	7.91^c^	0.264	8.00	9.61	0.187	<0.001	<0.001	<0.001	0.090	0.915	0.989	1.000
GE intake (MJ/day)^5^	168	157	170	158	4.9	148	178	3.5	0.173	0.034	<0.001	0.124	0.921	0.997	1.000
N^5^	0.20	0.18	0.19	0.19	0.006	0.17	0.21	0.004	0.157	<0.001	<0.001	<0.001	0.956	0.998	1.000
NDF^5^	4.16^b^	4.04^b^	3.94^b^	5.25^a^	0.128	3.96	4.74	0.090	<0.001	<0.001	<0.001	0.171	0.800	0.981	1.000
ADF^5^	2.60^b^	2.36^c^	2.37^c^	3.33^a^	0.080	2.42	2.91	0.056	<0.001	<0.001	<0.001	0.059	0.818	0.987	1.000
Oil^5^	0.32^b^	0.43^a^	0.31^b^	0.26^c^	0.010	0.30	0.36	0.007	<0.001	<0.001	<0.001	<0.001	0.223	0.963	1.000
EE^5^	0.24^b^	0.35^a^	0.23^b^	0.18^c^	0.007	0.22	0.27	0.005	<0.001	<0.001	<0.001	<0.001	0.106	0.973	1.000
Starch^5^	2.07^b^	1.72^c^	2.58^a^	-	0.064	1.93	2.32	0.052	<0.001	<0.001	<0.001	<0.001	0.697	0.999	0.993
WSC^5^	0.28^b^	0.16^d^	0.24^c^	0.61^a^	0.011	0.30	0.35	0.008	<0.001	<0.001	<0.001	<0.001	0.338	0.999	1.000
**Growth measurements**															
BW (kg)^6^	469^a^	465^a^	477^a^	426^b^	10.9	456	462	4.07	0.018	<0.001	0.705	<0.001	0.640	0.050	0.819
BWc (kg/day)^6^	1.42^a^	1.36^a^	1.58^a^	0.72^b^	0.136	0.81	1.72	0.096	<0.001	<0.001	<0.001	0.149	0.553	0.487	0.752
BWc/DMI (kg/kg)^6^	0.14	0.16	0.15	0.12	0.019	0.12	0.17	0.013	0.918	<0.001	0.008	0.013	0.966	0.776	0.398
**Enteric CH**_**4**_ **emissions**															
CH_4_ production (g/day)^7^	230^a^	213^a^	216^a^	167^b^	8.8	204	209	6.2	<0.001	0.024	0.603	0.068	0.157	0.091	0.930
CH_4_/DMI (g/kg)^7^	24.1^ab^	26.4^a^	22.5^bc^	19.6^c^	1.00	24.4	21.9	0.71	<0.001	<0.001	0.019	0.005	0.172	0.204	0.998
CH_4_/OMI (g/kg)^7^	25.4^ab^	27.5^a^	23.4^bc^	22.3^c^	0.99	26.3	22.9	0.70	0.006	<0.001	0.002	0.010	0.066	0.843	0.999
CH_4_/BW (g/kg)^6^	0.50^a^	0.44^ab^	0.46^a^	0.39^b^	0.020	0.44	0.46	0.014	0.011	<0.001	0.428	0.019	0.186	0.247	0.491
CH_4_/BWg (g/kg)^8^	256	204	251	187	33.3	235	180	23.8	0.345	0.003	0.155	0.471	0.123	0.116	0.337
CH_4_-E/GEI (MJ/MJ)^7^	0.08^a^	0.08^a^	0.07^a^	0.06^b^	0.003	0.079	0.069	0.002	<0.001	0.073	<0001	0.056	0.035	0.796	0.993

^1^SB = TMR including soy as the main protein source; BSG = TMR including local brewers’ spent grains as the main protein source; BNS = TMR including local field beans as the main protein source; GRA = diet containing 91:9 fresh-cut ryegrass:calf starter pellet as the GreenFeed bait.

^2^Significances were declared at *P* < 0.05. Significant differences between dietary treatments within variables are indicated with different superscript letters according to Fisher’s LSD test.

^3^DM = dry matter; OM = organic matter; ADF = acid detergent fiber; NDF = neutral detergent fiber; GE = gross energy; N = Nitrogen; BWg = body weight gain; CH_4_ = methane; DMI = DM intake; E = energy.

^4^Measurements for these variables were *n* = 144 for SB, *n* = 144 for BSG, *n* = 144 for BNS, and *n* = 141 for GRA.

^5^Measurements for these variables were *n* = 95 for SB, *n* = 95 for BSG, *n* = 96 for BNS, and *n* = 92 for GRA.

^6^Measurements for this variable were *n* = 104 for SB, *n* = 104 for BSG, *n* = 104 for BNS, and *n* = 104 for GRA.

^7^Measurements for this variable were *n* = 111 for SB, *n* = 99 for BSG, *n* = 108 for BNS, and *n* = 110 for GRA.

^8^For weeks where an animal has lost weight (BWc ≤ 0), the corresponding measurement was excluded from the dataset. Therefore, the number of measurements per treatment was *n* = 66 for SB, *n* = 65 for BSG, *n* = 72 for BNS, and *n* = 40 for GRA.

GRA had lower CH_4_ production (g/day) (by 46–63; 22.0–27.4%; *P *< 0.001) compared with the three concentrate-fed treatments and lower CH_4_/DMI (g/kg) (by 4.5 and 6.8; 18.7 and 25.8%; *P *< 0.001) compared with SB and BSG. CH_4_/OMI (g/kg) was higher (by 4.1 and 5.2; 14.9 and 18.9%; *P *= 0.006) for BSG compared with BNS and GRA and for SB compared with GRA (by 3.1–12.2%; *P *= 0.006). CH_4_/BW (g/kg) was higher (by 0.05–0.11; 11.4–22.0*%*; *P *= 0.011) for SB and BNS compared with GRA, while CH_4_ intensity (g/kg BW gain) did not differ (*P *> 0.05). CH_4_-E/gross energy intake (**GEI**; MJ/MJ) was higher for the three concentrate-fed treatments compared with GRA (*P *< 0.001). For feed and nutrient intakes, growth measurements, and enteric CH_4_ emissions, the effect of the week was significant (*P *< 0.05). A significant (*P *< 0.05) effect of Treatment × Week interaction was found for intakes of DM, N, oil, EE, Starch, and WSC (Supplementary Figure S1). Also, for BW (*P *< 0.001), BWc/DMI (*P *= 0.013), CH_4_ yield (g/kg DMI and g/kg OMI; *P *= 0.005 and *P *= 0.010, respectively), and CH_4_/BW (g/kg; *P *= 0.019). Regarding these significant interactions, GRA consistently had the lowest significance for BW across the experimental weeks, while its significance for BWc/DMI decreased over time (Figure 1). BSG consistently showed the highest significance across the weeks, indicating greater CH_4_ production per kg of DMI and OMI. In contrast, SB displayed the highest significance for CH_4_/BW throughout the experiment (Figure 2). At the start of the experiment, the CH_4_/BW for the SB treatment was higher; however, this difference decreased as the experiment progressed.

When the three concentrate-fed experimental treatments were compared after the GRA data were excluded from the dataset (as shown in Supplementary Table S1), the significant effect of treatment for CH_4_ yield (g/kg DMI and g/kg OMI) was similar as described above. The effect of the week was also similar as described above for the feed and nutrient intakes, growth measurements, and enteric CH_4_ emissions measurements. A significant effect of Treatment × Week interaction was found for ADF intake (*P *= 0.045), starch intake (*P *< 0.001), and WSC intake (*P *= 0.027). Across the whole experiment, starch intake was higher for BNS and lower for BSG, while WSC intake was higher for SB and again lower for BSG (Supplementary Figure S2).

### Feed and nutrient intakes and digestibility of steers during the chamber measurement periods

Feed and nutrient intakes (kg/day) and digestibility (kg/kg) from steers fed the four experimental diets during the chamber measurement periods of the animal trial are presented in Table 4 and for only the three concentrate-fed treatments in Supplementary Table S2. Intakes of DM, OM, GE, and N did not significantly differ (*P *> 0.05) among the dietary treatments. GRA supported higher (*P *= 0.032) NDF intake than SB and BNS, and higher (*P *= 0.015) ADF intake than BSG and BNS. Oil and EE intakes were higher (*P *< 0.001) for BSG, while starch intake was higher (*P *= 0.035) for BNS, and WSC intake was higher (*P *< 0.001) for GRA compared with the three concentrate-fed treatments. The Treatment × Period interaction effect was significant for starch (*P *= 0.014) and WSC (*P *< 0.001) intakes. Starch intake was higher for GRA compared with BSG for the chamber measurement periods 2, 3, and 4. In contrast, WSC intake was significant for GRA compared with the rest for the chamber measurement periods 1, 2, and 4 (Supplementary Figure S3). DM digestibility (*P *< 0.001), OM digestibility (*P *= 0.005), and digestible OM in DM (**DOMD**; *P *< 0.001) were lower for GRA, and DM digestibility was also lower (*P *< 0.001) for BSG compared with SB. NDF digestibility was lower (*P *= 0.012) for BSG than SB and for BNS than SB and GRA. For the four tested dietary treatments, the effect of the period was found to be significant in the feed and nutrient intakes (*P *< 0.05).

**Table 4.** Feed and nutrient intakes (kg/day) and digestibility (kg/kg) from steers fed the experimental diets during the chamber measurement periods of the animal trial

	Dietary treatments (D)^1^				SEM	*P*-values^2^		
Item^3^	SB (*n* = 16)	BSG (*n* = 16)	BNS (*n* = 15^4^)	GRA (*n* = 16)		D	Period (P)	D × P
**Feed and nutrient intakes (kg/day)**								
DM	5.71	5.84	5.67	5.14	0.357	0.544	<0.001	0.526
OM	5.41	5.56	5.41	4.67	0.472	0.558	<0.001	0.669
GE	96.3	105	96.5	92.7	8.65	0.771	0.005	0.722
N	113	120	105	115	14.9	0.908	0.016	0.494
NDF	2.38^b^	2.71^ab^	2.23^b^	3.06^a^	0.183	0.032	<0.001	0.867
ADF	1.49^b^	1.59^b^	1.32^b^	1.94^a^	0.117	0.015	0.004	0.833
Oil	0.18^b^	0.29^a^	0.18^b^	0.15^b^	0.017	<0.001	0.001	0.917
EE	0.14^b^	0.23^a^	0.13^b^	0.11^b^	0.012	<0.001	0.004	0.984
Starch	1.18^b^	1.15^b^	1.46^a^	-	0.063	0.035	0.007	0.014
WSC	0.17^b^	0.11^b^	0.14^b^	0.35^a^	0.021	<0.001	0.002	<0.001
**Digestibility (kg/kg)**								
DM	0.70^a^	0.66^b^	0.68^ab^	0.56^c^	<0.001	<0.001	0.200	0.216
OM	0.73^a^	0.69^a^	0.71^a^	0.58^b^	0.005	0.005	0.127	0.492
DOMD	0.69^a^	0.65^a^	0.67^a^	0.53^b^	<0.001	<0.001	0.291	0.526
N	0.59	0.61	0.53	0.55	0.078	0.078	0.076	0.750
NDF	0.64^a^	0.59^bc^	0.57^c^	0.61^ab^	0.012	0.012	0.210	0.389
ADF	0.57	0.52	0.47	0.52	0.219	0.219	0.270	0.617

^1^SB = TMR including soy as the main protein source; BSG = TMR including local brewers’ spent grains as the main protein source; BNS = TMR including local field beans as the main protein source; GRA = diet including solely fresh-cut ryegrass.

^2^Significances were declared at *P* < 0.05. Significant differences between dietary treatments within variable are indicated with different superscript letters according to Fisher’s LSD test.

^3^DM = dry matter; OM = organic matter; N = nitrogen; NDF = neutral detergent fiber; ADF = acid detergent fiber; EE = ether extract; WSC = water soluble carbohydrates; DOMD = digestible OM in DM; GE = gross energy.

^4^There was a missing measurement in one animal in the BNS treatment in the last period of the experiment.

When the three concentrate-fed experimental treatments were compared after the GRA data were excluded from the dataset (as shown in Supplementary Material), the significant effect of treatment for oil, EE, and starch intakes and the Treatment × Period interaction effect were similar as described above, while WSC intake for SB was higher (*P *= 0.016) for SB compared with BSG. DM digestibility and DOMD were found lower (*P *= 0.004 and *P *= 0.006, respectively) for BSG compared with SB. In addition, OM digestibility was lower (*P *= 0.004) for BSG compared with SB and BNS and for BNS compared with SB. N digestibility was higher (*P *= 0.010) for BSG compared with BNS. The effect of the period was significant only for the N digestibility (*P *= 0.040).

### Energy metabolism and enteric CH_4_ emissions from steers during the chamber measurement periods

Energy metabolism and enteric CH_4_ emissions from steers fed the four experimental diets during the chamber measurement periods of the animal trial are presented in Table 5 and from steers fed the three concentrate-fed treatments only in Supplementary Table S3. Fecal energy output was the highest (*P *= 0.007) for GRA and BSG, while urine E energy output was higher (*P *< 0.001) for GRA than for the three concentrate-fed treatments. GRA had the lowest CH_4_-E output (*P *< 0.001). As for energy utilization, DE/GE (*P *< 0.001), ME/GE (*P *< 0.001), and ME/DE (*P *= 0.001) were lower for GRA, while fecal and urine E outputs/GEI were higher (*P *< 0.001) for GRA when compared with the three concentrate-fed treatments.

**Table 5.** Energy metabolism and enteric CH_4_ emissions from steers fed the experimental diets during the chamber measurement periods of the animal trial

Item^3^	Dietary treatments (D)^1^				SEM	*P*-values^2^		
	SB (*n* = 16)	BSG (*n* = 16)	BNS (*n* = 15^4^)	GRA (*n* = 16)		D	Period (P)	D × P
**Energy intakes and outputs (MJ/day)**								
GE intake	96.3	105	96.5	92.7	8.65	0.771	0.005	0.722
Fecal E output	31.5^b^	38.1^a^	31.5^b^	40.8^a^	1.71	0.007	<0.001	0.110
Urine E output	2.26^b^	2.76^b^	2.11^b^	4.76^a^	0.235	<0.001	0.360	0.409
CH_4_-E output	13.4^a^	13.0^a^	12.8^a^	8.91^b^	0.455	<0.001	<0.001	0.511
DE intake	64.8	67.2	63.1	46.4	6.59	0.222	0.084	0.456
ME intake	61.8	63.7	60.2	41.5	6.55	0.174	0.094	0.409
**Energy utilization (MJ/MJ)**								
DE/GE	0.67^a^	0.64^a^	0.65^a^	0.52^b^	0.020	<0.001	0.199	0.231
ME/GE	0.64^a^	0.61^a^	0.62^a^	0.48^b^	0.013	<0.001	0.545	0.391
ME/DE	0.95^a^	0.95^a^	0.96^a^	0.87^b^	0.011	0.001	0.327	0.075
Fecal E output/GEI	0.33^b^	0.36^b^	0.36^b^	0.45^a^	0.016	<0.001	0.451	0.665
Urine E output/GEI	0.024^b^	0.026^b^	0.022^b^	0.052^a^	0.003	<0.001	0.136	0.314
**Enteric CH**_**4**_ **emissions**								
CH_4_ production (g/day)	241^a^	234^a^	230^a^	160^b^	7.41	<0.001	<0.001	0.514
CH_4_/DMI (g/kg)	42.7^a^	40.1^a^	41.1^a^	32.7^b^	1.37	<0.001	0.663	0.902
CH_4_/OMI (g/kg)	45.1^a^	42.1^a^	43.1^a^	36.2^b^	1.76	0.010	0.683	0.717
CH_4_/digestible DMI (g/kg)	60.9	61.2	61.1	70.0	4.62	0.449	0.235	0.220
CH_4_/digestible OMI (g/kg)	62.1	61.6	72.5	61.2	5.39	0.428	0.339	0.730
CH_4_/BW (g/kg)	0.50^a^	0.51^a^	0.47^a^	0.37^b^	0.017	0.001	0.012	0.278
CH_4_-E/GEI (MJ/MJ)	0.14^a^	0.12^a^	0.13^a^	0.10^b^	0.007	0.006	0.713	0.952
CH_4_-E/DEI (MJ/MJ)	0.21	0.20	0.21	0.25	0.026	0.441	0.175	0.198
CH_4_-E/MEI (MJ/MJ)	0.22	0.21	0.22	0.30	0.032	0.172	0.227	0.221
**Other respirometry measurements**								
CO_2_ production (L/day)	4012^a^	4103^a^	4182^a^	3254^b^	135.9	0.003	0.176	0.373
O_2_ production (L/day)	4129^a^	4180^a^	4148^a^	3309^b^	96.3	<0.001	0.058	0.344
Heat production (MJ/day)	85.7^a^	86.3^a^	86.3^a^	68.5^b^	2.012	<0.001	0.057	0.283
Respiratory Quotient (L/day)	1.00^ab^	0.98^bc^	1.01^a^	0.99^b^	0.006	0.030	0.923	0.942

^1^SB = TMR including soy as the main protein source; BSG = TMR including local brewers’ spent grains as the main protein source; BNS = TMR including local field beans as the main protein source; GRA = diet including solely fresh-cut ryegrass.

^2^Significances were declared at *P* < 0.05. Significant differences between dietary treatments within variable are indicated with different superscript letters according to Fisher’s LSD test.

^3^GE = gross energy; E = energy; CH_4_ = methane; DE = digestible energy; ME = metabolizable energy; DEI = DE intake; MEI = ME intake.

^4^There was a missing measurement in one animal in the BNS treatment in the last period of the experiment.

When compared with the three concentrate-fed treatments GRA showed lower CH_4_ production (by 70–81 g/day; 30–34%; *P *< 0.001), CH_4_/DMI (by 7.4–10.0 g/kg; 18–23%; *P *< 0.001), CH_4_/OMI (by 5.9–8.9 g/kg; 14.0–19.7%; *P *= 0.010), CH_4_-E/GEI (by 0.2–0.4 MJ/MJ; 17–29%; *P *= 0.006), and CH_4_/BW (by 0.10–0.14 g/kg; 21.3–27.5%; *P *= 0.001). As for other respiratory measurements, CO_2_ (L/day; *P *= 0.003) and O_2_ (L/day; *P *< 0.001) productions, as well as heat production (MJ/day) were the lowest (*P *< 0.001) for GRA compared with the three concentrate-fed treatments; by 758–928 L/day (18.9–22.2%), 820–871 L/day (19.9–20.8%), and 17.2–17.8 MJ/day (20.1–20.6%), respectively. The effect of the period was found significant for GEI (MJ/day; *P *= 0.005), fecal E output (MJ/day; *P *< 0.001), CH_4_-E output (MJ/day; *P *< 0.001), CH_4_ production (g/day; *P *< 0.001), and CH_4_/BW (g/kg; *P *= 0.012).

When the three concentrate-fed experimental treatments were compared after the GRA data were excluded from the dataset (as shown in Supplementary Material), there were no significant differences in energy intakes and outputs, energy utilization, enteric CH_4_ emissions, and other respirometry measurements. Significant (*P* < 0.05) effects of Treatment × Period interactions were found for the other respiratory measurements; however, the pairwise comparisons did not show any significant effect in different weeks across periods 1–4 (Supplementary Figure S4).

### Nitrogen intake and outputs and nitrogen utilization from steers during the chamber measurement periods

Results regarding NI and N outputs (g/day) and nitrogen utilization (g/g) are presented in Table 6 and from steers fed only the three concentrate-fed treatments in Supplementary Table S4. There was no significant (*P *> 0.05) treatment effect for NI, FNO, and FNO/NI. When compared with the other three concentrate-fed treatments, UNO and MNO were higher for GRA (by 56–77.3 g/day, *P *= 0.026; and by 53–73.4 g/day *P *= 0.034, respectively), while retained N was lower (by 58.4–62.0 g/day*, P *= 0.028). Regarding N utilization parameters, UNO/NI (*P *= 0.002) and MNO/NI (*P *= 0.006) were the highest, while retained N/NI was the lowest (*P *= 0.008) for GRA. In addition, FNO/MNO was higher (*P *= 0.039) for BNS and SB compared with GRA. UNO/MNO were higher (*P *= 0.039) for GRA compared with SB and BNS and UNO/FNO was higher (*P *= 0.010) for GRA compared with the three concentrate-fed treatments. The effect of the period was significant for NI (*P *= 0.016), feces N output (*P *< 0.001), feces N output/NI (*P *= 0.001), and UNO/FNO (*P *= 0.031). As for the interaction effect of Treatment × Period, this was found significant only for retained N/NI (*P *= 0.027), which was found to be lower across the chamber measurement periods for GRA compared with the concentrate-based treatments (Supplementary Figure S3).

**Table 6.** Nitrogen intake and outputs (g/day) and nitrogen utilization (g/g) from steers fed the experimental diets during the chamber measurement periods of the animal trial

Item^3^	Dietary treatments (D)^1^				SEM	*P*-values^2^		
	SB (*n* = 16)	BSG (*n* = 16)	BNS (*n* = 15^4^)	GRA (*n* = 16)		D	Period (P)	D × P
**N intakes and outputs (g/day)**								
N intake	113	120	105	115	14.9	0.908	0.016	0.494
Feces N output	45.8	46.9	48.2	44.4	2.78	0.794	<0.001	0.171
Urine N output	57.8^b^	66.8^b^	45.5^b^	122.8^a^	13.55	0.026	0.175	0.464
Manure N output	104^b^	114^b^	93.6^b^	167^a^	15.2	0.034	0.230	0.559
Retained N	9.58^a^	6.21^a^	9.75^a^	−52.2^b^	13.65	0.028	0.846	0.722
**N utilization (kg/kg)**								
Feces N output/NI	0.41	0.39	0.46	0.45	0.036	0.437	0.001	0.105
Urine N output/NI	0.52^b^	0.56^b^	0.45^b^	1.21^a^	0.113	0.002	0.272	0.255
Manure N output/NI	0.93^b^	0.95^b^	0.92^b^	1.66^a^	0.138	0.006	0.228	0.209
Retained N/NI	0.45^a^	0.30^a^	0.48^a^	−4.16^b^	0.866	0.008	0.184	0.027
FNO/MNO	0.45^a^	0.41^ab^	0.54^a^	0.29^b^	0.038	0.039	0.129	0.510
UNO/MNO	0.55^b^	0.59^ab^	0.46^b^	0.71^a^	0.038	0.039	0.129	0.510
UNO/FNO	1.29^b^	1.43^b^	1.01^b^	2.88^a^	0.319	0.010	0.031	0.188

^1^SB = TMR including soy as the main protein source; BSG = TMR including local brewers’ spent grains as the main protein source; BNS = TMR including local field beans as the main protein source; GRA = diet including solely fresh-cut ryegrass.

^2^Significances were declared at *P *< 0.05. Significant differences between dietary treatments within variable are indicated with different superscript letters according to Fisher’s LSD test.

^3^N = nitrogen, NI = N intake; FNO = feces N output; MNO = manure N output; UNO = urine N output.

^4^There was a missing measurement in one animal in the BNS treatment in the last period of the experiment

When the three concentrate-fed experimental treatments were compared after the GRA data were excluded from the dataset (as shown in Supplementary Material), MNO was significantly higher for BSG compared with SB and BNS (by 10–21.1 g/day, *P *< 0.001) and for SB compared with BNS (by 11.1 g/day, *P *< 0.001). Furthermore, feces N output/NI was higher for BNS than for BSG (by 7 g/day, *P *= 0.009).

### Effect of sex on feed and nutrient intakes, growth measurements, and enteric CH_4_ emissions

Steers had higher intakes of DM (by 1.48 kg/day; *P *< 0.001), OM (by 1.61 kg/day; *P *< 0.001), GE (by 30 MJ/day; *P *< 0.001), N (by 0.04 kg/day; *P *< 0.001), ADF (by 0.49 kg/day; *P *< 0.001), NDF (by 0.78 kg/day; *P *< 0.001), oil (by 0.06 kg/day; *P *< 0.001), EE (by 0.05 kg/day; *P *< 0.001), starch (by 0.39 kg/day; *P *< 0.001), and WSC (by 0.05 kg/day; *P *< 0.001), when compared with heifers (Table 1). They also had higher average BWc (by 0.91 kg/day; *P *< 0.001) and BWc/DMI (by 0.05 kg/kg; *P* = 0.008). When compared with heifers, steers had lower CH_4_ yield (by 2.5 and 3.4 g/kg DMI and g/kg OMI, *P *= 0.019 and *P *= 0.002, respectively) and CH_4_-E/GEI (by 0.01 MJ/MJ; *P *< 0.001). A significant (*P* = 0.035) effect of Treatment × Sex interaction was found for CH_4_-E/GEI, with BSG heifers having higher yield compared with SB and GRA, and the steers of the three concentrate-based treatments having higher yield compared with GRA (Figure 2).

When the three concentrate-fed experimental treatments were compared after the GRA data were excluded from the dataset (as shown in Supplementary Material), the significant effect of sex for feed and nutrient intakes, growth measurements, and enteric CH_4_ emissions were similar as described above. A significant effect of Treatment × Sex interaction was found for CH_4_/OMI (g/kg; *P *= 0.025) and CH_4_-E/GEI (MJ/MJ; *P* = 0.016), with BSG demonstrating higher yield compared with SB and BNS. Regarding steers, SB and BNS had higher CH_4_/OMI compared with BNS, and SB had higher CH_4_-E/GEI compared with BNS (Supplementary Figure S5).

## Discussion

Utilizing locally sourced feeds and byproducts reduces reliance on imported feedstuffs linked with sustainability-related issues and high transportation costs, such as soybean, thus, may alleviate environmental burdens (Herrero et al., 2016; Pexas et al., 2023), improving resource efficiency toward a more sustainable and overall resilient livestock sector. This study evaluated the effect of replacing soybean meal with two different local UK protein sources on nutrient utilization, environmental outputs (CH_4_ emissions, N excretion), and animal performance, and compared these systems against a pasture-based system.

### Comparison between dietary protein sources

#### Nutrient intakes and utilization

Fiber and more specifically NDF is the main component in rations that reduces intake and digestibility (Mertens, 2009). A meta-analysis based on growing ruminants (cattle, sheep, and goats) revealed a negative linear relationship between NDF and DMI, and OM digestibility (Salah et al., 2015). In our study, DMI, OM digestibility, and DOMD were significantly reduced for BSG. In addition, oil and EE intakes were greater for BSG compared with SB and BNS. In addition, dietary fat may hinder fiber digestion (Beauchemin et al., 2007; Patra, 2013), which could partially explain the decreased NDF digestibility for BSG compared with SB. Regarding the use of field beans as a replacement for soy, antinutritional factors such as tannins, trypsin, protease inhibitors, and phytoestrogens could reduce intake and digestibility (Dvořák et al., 2006; Huang et al., 2018; Johnston et al., 2019). However, when field beans replaced rapeseed meal (Puhakka et al., 2016) or concentrate ingredients (Johnston et al., 2019) in dairy cow diets, intake and digestibility were not significantly affected. In that case, the rapeseed-based diet may have already been of reduced digestibility due to goitrogens and glucosinolates, which can be significant antinutritional factors present in rapeseed meal (Bischoff, 2021). Dairy cows and growing steers have different nutritional requirements and physiological statuses, and thus, results in dairy cows may not extrapolate to beef cattle (McDonald et al., 2010).

Increasing the dietary supply of rumen-degradable grain starch could also decrease fiber digestion (Poore et al., 1993; Martin et al., 1999). Compared with SB, BNS had higher starch content (259 vs. 207 g/kg DM) and starch intake (1.46 vs. 1.18 kg/day) and had lower NDF and ADF digestibility.

#### Energy metabolism and enteric CH_4_ emissions

Comparing the three concentrate-fed treatments (SB vs. BSG vs. BNS), there were no significant differences in energy utilization and enteric CH_4_ emission parameters during chamber measurement periods. However, during the group-housing weeks, we found a higher CH_4_ yield (g/kg DMI and g/kg OMI) for BSG compared with BNS. Although both DMI and NDF content are positively related to beef cattle CH_4_ production (Ellis et al., 2007; Yan et al., 2009), compared with SB and BNS, BSG had lower DMI and OMI in the group-housed period for growing beef (steers and heifers). Lower DMI is associated with longer rumen retention, leading to more CH_4_ yield. Additionally, SB and BNS had higher starch content than BSG and increased starch will support propionate production and result in reduced enteric CH_4_ production and yield (Bannink et al., 2006; Hatew et al., 2015). However, previous research has shown that the partial substitution of wheat grain and solvent-extracted canola meal by brewers’ grains (259 g/kg DM) significantly reduced dairy cows’ CH_4_ yield by 5.2% (g/kg DMI), and CH_4_ intensity by 9.05% (g/L milk) (Moate et al., 2011). Furthermore, replacing grass silage with brewers’ grains in barley straw-based diets, in pregnant nonlactating beef cows, reduced CH_4_ yield (g/kg DMI) by up to 22.8% (Duthie et al., 2015). When comparing results across studies, it is essential to consider factors such as the inclusion level of the test feed, the type of feed being replaced in the diet, variability in diet composition, and the characteristics of the basal diet into which the test feed is introduced. For example, brewers’ grains were included at 259 and 226 g/kg DM in the studies by Moate et al. (2011) and Duthie et al. (2015), respectively, while our study used a lower inclusion level of 183 g/kg DM. Moreover, the present study replaced soybean meal with brewers’ spent grains, whereas other studies replaced cracked wheat grain and solvent-extracted canola meal (Moate et al., 2011) or grass silage (Duthie et al., 2015).

To our knowledge, no studies have specifically evaluated the impact of field beans on nutrient and energy metabolism, as well as enteric CH_4_ emissions, in growing beef cattle. However, similar to the present study (Cherif et al., 2018) found that replacing soybean meal with fava beans as an alternative protein source in dairy cow diets did not significantly affect enteric CH_4_ production. This was likely because fava beans did not sufficiently alter the protein and NDF balance when replacing soybean meal and soy hulls. Therefore, the absence of a significant effect on starch concentration may have limited any potential influence on enteric CH_4_ emissions.

#### Nitrogen utilization efficiency

Concerning the effect of the different protein sources on N utilization, it is important to note that NI did not differ between the three concentrate-fed groups. The numerically higher UNO for BSG compared with SB and BNS (trend at *P* = 0.071; Supplementary Table S4), have resulted in the increased MNO, given that FNO was similar between the three treatments. In addition, the FNO/NI was lower for BSG compared with SB and BNS, which may indicate a slight diversion of N output from feces to urine when BSG was fed. Although the diets were considered isonitrogenous and were formulated to provide similar amounts of metabolizable protein and effective ruminal degradable protein (**RDP**), the shift in N excretion may be attributable to the higher rapidly RDP content that may be due to the brewers’ spent grains compared with the less rapidly degradable protein in soybean or field beans. Higher rapid RDP can increase the likelihood of *n* being converted to urea and ammonia (**NH**_**3**_), which are then diverted to urine excretion (Hoekstra et al., 2007).

Replacing soybean and corn grain with fava beans also did not affect N output in dairy cows (Cherif et al., 2018). Overall, this potential shift in N excretion from feces to urine may be considered environmentally undesirable, given that urinary N is a main source of N_2_O emissions from livestock systems (Dijkstra et al., 2013).

### Comparison between concentrate- and pasture-based diet

#### Nutrient intakes and utilization

DMI was reduced by 41.6%, 31.3%, 41.8%, and 40.6% for SB, BSG, BNS, and GRA, respectively, when animals were housed in the respiration chambers compared with when they were group-housed in the barn. Previous research also reported a 14.9% reduction in DMI in steers isolated in respiration chambers compared with when group-housed (Llonch et al., 2018). Treatment did not affect DMI while animals were in chambers, but DMI was higher for SB and BNS (9.78 and 9.74 kg/day, respectively) compared with BSG (8.50 kg/day) and GRA (8.65 kg/day) when animals were group-housed. This may be attributed to the higher NDF content for BSG TMR and fresh-cut ryegrass, which is known to reduce palatability, digestibility, and feed intake in cattle (Mertens, 2009). When animals were in respiration chambers, physical fill effects on DMI were overridden by other factors such as behavioral, affecting DMI. Additionally, the increased NDF and ADF intake contributed to a reduction in DM digestibility and DOMD in steers confined to respiration chambers. Significant differences were also found in OMI that could be attributed to the varied nutritional composition of the diets, with the fresh-cut ryegrass having the lowest nutritional density (i.e., lowest OM and starch content). Mertens (2009) highlighted that higher OMI often correlates with improved nutrient digestibility and energy availability, which aligns with the results of the present study, which found lower DM digestibility, DOMD, and GE digestibility in the pasture-fed compared with the concentrate-fed steers. The lower OM content for GRA resulted in a lower OM intake for beef in group-housed periods. More specifically, for the group-housed beef, OMI was the highest for BNS (9.58 kg/day) and lowest for GRA (7.91 kg/day). Furthermore, GRA beef had the highest ADF and NDF intakes, which is consistent with the high fiber content typically found in grass-based diets.

Although feed efficiency (BWc/DMI) did not significantly differ overall between the treatments, BSG and BNS had numerically higher efficiency by 12.5% and 6.7%, respectively, compared with SB. The three concentrate-fed treatments had also numerically higher efficiency (BWc/DMI) than GRA by 14.3% (SB), 25% (BSG), and 20% (BNS), respectively. Differences in feed efficiency have been however statistically significant different within specific measurement weeks; for example, although they were higher in GRA in week 2, they ended up being lower for GRA than the three concentrate-fed treatments in most cases between weeks 7 and 12, which may indicate that GRA diet may have been less efficient as the animals were growing older and heavier. Therefore, despite the likely higher nutritional value of meat, with greater concentrations of unsaturated and lower concentrations of saturated fatty acids from a forage-based system (Średnicka-Tober et al., 2016; Ribas-Agustí et al., 2019; Clinquart et al., 2022; Klopatek et al., 2022), the potential risk to productivity should be considered when implementing high-forage diets, which are typically used to reduce production costs (Berthiaume et al., 2006; Santos-Silva et al., 2023).

#### Energy metabolism and enteric CH_4_ emissions

Despite the nonsignificant differences in GE intakes, feeding fresh-cut ryegrass to steers resulted in higher fecal and urine energy output, leading to lower digestibility and energy use efficiency. Forage-based diets could lead to greater fecal energy losses due to the high fiber content, which is itself less digestible compared with concentrate-based diets (Beauchemin et al., 2008; McDonald et al., 2010), leading to reduced energy utilization (Waghorn and Clark, 2004). This is also supported in this study by the lower DE/GE and ME/GE ratios in GRA beef.

The average CH_4_ production across treatments during the chamber measurement periods was 215 ± 44 g/day, which is consistent with literature findings reporting a range of 161 ± 20 g/day for steers of reduced average weight (325 ± 20 kg) compared with the present study (Laubach et al., 2008) and up to on average 323 g/day for beef cattle (Broucek, 2014). Nevertheless, different conditions and diet management practices can influence CH_4_ production outcomes in various ways. When evaluating the effect of pasture on CH_4_ emissions, forage quality should also be accounted for. High-quality pasture, particularly during the early grazing season, can reduce CH_4_ emissions by 44% and 29% in steers compared with pasture during the mid and late grazing seasons, respectively (Boadi, 2004). Furthermore, DMI and NDF content could be positively related to beef cattle CH_4_ production (MJ/day and L/day) (Ellis et al., 2007; Yan et al., 2009). In the present study, DMI did not differ for steers in chambers between the four dietary treatments but was numerically lower for GRA. Despite the fact that GRA had the higher NDF content and NDF intake, GRA steers resulted in lower CH_4_ production (g/day) and CH_4_ yield (g/kg DMI and g/kg OMI). Increased dietary NDF may not always result in higher CH_4_ production due to the presence of lignin, which is indigestible and reduces the fermentability of NDF (van Lingen et al., 2019). Lower daily CH_4_ production (g/day) and CH_4_ yield (g/kg DMI and g/kg OMI) for GRA could be attributed to the lower fermentable carbohydrate availability in fresh-cut ryegrass. Higher fiber diets, while leading to higher CH_4_ yield per unit of intake, can also reduce overall CH_4_ production (g/d) due to lower total feed intake and digestibility (Johnson and Johnson, 1995). Increased digestibility (OM or GE) has been associated with increased CH_4_ yield per unit of DMI or GE (Ramin and Huhtanen, 2013). This is consistent with the present study, which found higher CH_4_ yield (g/kg DMI and g/kg OMI) for the three concentrate-based diets compared with the fresh-cut ryegrass-fed steers, which also had lower DM, OM, DOMD, and GE digestibility. Therefore, the fact that CH_4_ yield (g/kg digestible DMI and g/kg digestible OMI) was not significantly affected, could further support that digestibility was amongst the main drivers for the reduction of enteric CH_4_ emissions for pasture-based diets. However, considering CH_4_ emissions in relation to cattle performance, with a starting BW of 394 kg and a target slaughter weight of 550 kg, GRA cattle would require 184 d to reach slaughter, compared with 110, 115, and 99 d for SB, BSG, and BNS cattle, respectively. Consequently, lifetime CH_4_ production per kg of meat is higher for GRA cattle at 30.6 kg, compared with 25.3 kg (SB), 24.4 kg (BSG), and 21.3 kg (BNS), respectively.

#### Nitrogen utilization efficiency

Improving N use efficiency (Calsamiglia et al., 2010) can have economic benefits (reduced use of N in the diet) and reduces nitrogenous emissions and N leaches in the groundwater (Calsamiglia et al., 2010; Dijkstra et al., 2013). Studies have shown that 1 g of NI can increase UNO and FNO by 0.51 and 0.20 g in beef cattle being in different maturity stages (growing, finishing, and mature) and under different diets with varying forage inclusion rates, respectively (Dong et al., 2014). In the present study, the higher UNO, UNO/NI, MNO/NI, and the negative retained N/NI for GRA steers suggest a lower N use efficiency, with the main excretion pathway being through urine. Negative N retention in the pasture-fed steers further supports lower N use efficiency. Studies have shown that there is a positive correlation between the total fiber in the diet with manure N excretion in beef cattle (Yan et al., 2007). This is likely because forages and grazed grass are rich in rapidly degradable protein and nonprotein N and the fibrous structural carbohydrates are fermented at slow rates (causing delays in energy supply for microbial synthesis) (Hoekstra et al., 2007). As a result, there is an extensive synthesis of NH_3_ and urea, part of which is then diverted to urine for excretion (Weiss et al., 2003; Tas et al., 2006; Hoekstra et al., 2007). The higher MNO could further increase the N loss in GRA steers. In line with previous studies, GRA steers had a lower FNO/MNO ratio (0.29 kg/kg) than the UNO/MNO ratio (0.71 kg/kg), showing that the main pathway for excess N excretion is through urine. Consequently, the results of the present study indicate that forage-based low-input diets could lead to higher N losses and reduced N utilization than diets containing 36% concentrate.

### Effect of sex on nutrient balance and enteric CH_4_ emissions in growing beef

The lower feed and nutrient intakes in heifers compared with steers are consistent with Owens and Gardner (2000), who found that steers generally exhibit higher feed intakes and growth rates. Interestingly, in the present study, heifers had higher CH_4_ yield compared with steers, while Thompson et al. (2019) found that heifers produced less than steers. Furthermore, the effectiveness of dietary treatments in reducing CH_4_ emissions may vary by sex and diet, with ryegrass-fed heifers showing reduced CH_4_ (CH_4_-E/GEI) only compared with BSG-heifers, while in steers, this reduction was observed compared with the rest treatments also. The present study also suggests that the effectiveness of alternative protein sources in reducing CH_4_ emissions may depend on sex. For example, replacing soybean with field beans appears to reduce CH_4_ emissions in steers, but not in heifers. Conversely, replacing soybean with brewers’ spent grains may increase CH_4_ emissions in heifers, but not in steers. Beyond the practical applications and impact this may have on farm-level emissions, this indicates the importance of the efficacy of CH_4_ mitigation via dietary changes to account for both sexes in experimental work. This interaction was not significant for the intake and growth variables, indicating that both heifers and steers responded similarly to the dietary treatments; a finding that may improve the applicability of the proposed feeding practices across both sexes.

## Conclusion

Diets containing protein sources with low fiber or high starch, such as soy and field beans, promote better growth performance and nutrient utilization compared with more fibrous diets, such as brewers’ spent grains and fresh-cut ryegrass. The study showed that field beans can be included in growing beef diets up to 15.6% DM, replacing soybean meal and soy hulls, without affecting productivity and environmental outputs from the animal (enteric methane emissions, nitrogen excretion). The higher fiber content in the diets of beef-fed fresh-cut Italian ryegrass with minimal concentrate (91:9 ratio) reduced methane production and methane yield per kg of DM and OMI compared with concentrate-based fed beef, with no effect on methane intensity, but increased nitrogen excretion in feces and urine. The pasture-based diet also reduced growth rate and feed efficiency (gain-to-feed ratio), traits that should be taken into consideration when this feeding strategy is followed to reduce production costs.

## Supplementary Material

skaf007_suppl_Supplementary_Material

## Data Availability

The data that support the findings of this study are available from the corresponding author, upon reasonable request.

## Abbreviations

ADF: acid detergent fiber
BNS: total mixed ration including local field beans as the main protein source
BSG: total mixed ration including local brewers’ spent grains as the main protein source
BW: body weight
BWc: body weight change
BWg: body weight gain
CP: crude protein
DE: digestible energy
DEI: digestible energy intake
DM: dry matter
DMI: dry matter intake
DOMD: digestible organic matter in dry matter
EE: ether extract
FNO: fecal nitrogen output
GE: gross energy
GEI: gross energy intake
GRA: diet containing solely fresh-cut ryegrass
ME: metabolizable energy
MEI: metabolizable energy intake
MNO: manure nitrogen output
NI: nitrogen intake
NDF: neutral detergent fiber
OM: organic matter
OMI: organic matter intake
SB: total mixed ration including soybean meal as the main protein source
UNO: urinary nitrogen output
WSC: water soluble carbohydrates

## References

[CIT0001] AOAC. 2012. Official methods of analysis of the Association of Official Analytical Chemists. 19th ed. Washington (DC): Association of Official Analytical Chemists.

[CIT0002] Bannink, A., J. Kogut, J. Dijkstra, J. France, E. Kebreab, A. M. Van Vuuren, and S. Tamminga. 2006. Estimation of the stoichiometry of volatile fatty acid production in the rumen of lactating cows. J. Theor. Biol. 238:36–51. doi: https://doi.org/10.1016/j.jtbi.2005.05.026 16111711

[CIT0004] Beauchemin, K. A., S. M. McGinn, and H. V. Petit. 2007. Methane abatement strategies for cattle: lipid supplementation of diets. Can. J. Anim. Sci. 87:431–440. doi: https://doi.org/10.4141/cjas07011

[CIT0003] Beauchemin, K. A., M. Kreuzer, F. O’Mara, and T. A. McAllister. 2008. Nutritional management for enteric methane abatement: a review. Aust. J. Exp. Agric. 48:21–27. doi: https://doi.org/10.1071/ea07199

[CIT0005] Berthiaume, R., I. Mandell, and L. Faucitano. and C. Lafrenière. 2006. Comparison of alternative beef production systems based on forage finishing or grain-forage diets with or without growth promotants: 1. Feedlot performance, carcass quality, and production costs. J. Anim. Sci. 84: 2168–2177. doi: https://doi.org/10.2527/jas.2005-328 16864879

[CIT0006] Bischoff, K. L. 2021. Chapter 53 - Glucosinolates. In: Gupta R. C., R. Lall, and A. Srivastava, editors. Nutraceuticals. 2nd ed. Amsterdam, (Netherlands): Elsevier Science; p. 903–909.

[CIT0007] Boadi, D. A. 2004. Field measurement of methane and carbon dioxide production by cattle: use of the sulphur hexafluoride (SF/4) tracer gas technique. Canada: University of Manitoba, Winnipeg, MB (Canada); Manitoba Univ., Winnipeg, MB (Canada).

[CIT0008] Boval, M., and R. M. Dixon. 2012. The importance of grasslands for animal production and other functions: a review on management and methodological progress in the tropics. Animal 6:748–762. doi: https://doi.org/10.1017/S1751731112000304 22558923

[CIT0009] Broucek, J. 2014. Production of methane emissions from ruminant husbandry: a review. J. Environ. Prot. 05:1482–1493. doi: https://doi.org/10.4236/jep.2014.515141

[CIT0010] Calsamiglia, S., A. Ferret, C. K. Reynolds, N. B. Kristensen, and A. M. van Vuuren. 2010. Strategies for optimizing nitrogen use by ruminants. Animal 4:1184–1196. doi: https://doi.org/10.1017/S1751731110000911 22444616

[CIT0011] Cherif, C., F. Hassanat, S. Claveau, J. Girard, R. Gervais, and C. Benchaar. 2018. Faba bean (*Vicia faba*) inclusion in dairy cow diets: effect on nutrient digestion, rumen fermentation, nitrogen utilization, methane production, and milk performance. J. Dairy Sci. 101:8916–8928. doi: https://doi.org/10.3168/jds.2018-14890 30100504

[CIT0012] Clinquart, A., M. P. Ellies-Oury, J. F. Hocquette, L. Guillier, V. Santé-Lhoutellier, and S. Prache. 2022. Review: on-farm and processing factors affecting bovine carcass and meat quality. Animal 16:100426. doi: https://doi.org/10.1016/j.animal.2021.100426 35031228

[CIT0013] de Visser, C. L. M., R. Schreuder, and F. Stoddard. 2014. The EU’s dependency on soya bean import for the animal feed industry and potential for EU produced alternatives. Ocl 21:D407. doi: https://doi.org/10.1051/ocl/2014021

[CIT0014] Dijkstra, J., O. Oenema, J. W. van Groenigen, J. W. Spek, A. M. van Vuuren, and A. Bannink. 2013. Diet effects on urine composition of cattle and N2O emissions. Animal 7:292–302. doi: https://doi.org/10.1017/S1751731113000578 23739471

[CIT0015] Dong, R. L., G. Y. Zhao, L. L. Chai, and K. A. Beauchemin. 2014. Prediction of urinary and fecal nitrogen excretion by beef cattle. J. Anim. Sci. 92:4669–4681. doi: https://doi.org/10.2527/jas.2014-8000 25149338

[CIT0016] Duthie, C. A., J. A. Rooke, J. J. Hyslop, and A. Waterhouse. 2015. Methane emissions from two breeds of beef cows offered diets containing barley straw with either grass silage or brewers’ grains. Animal 9:1680–1687. doi: https://doi.org/10.1017/S1751731115001251 26145179

[CIT0017] Dvořák, R., A. Pechová, L. Pavlata, B. Klejdus, K. Kovařčík, J. Dostálová, J. Culková, J. Filípek, E. Svajdlenka, and V. Capkova. 2006. Reduction in the content of antinutritional substances in Fava beans (*Vicia faba*) by different treatments. In: Zemljič B., editor. 7th Middle European buiatric congress 43:174–179. Radenci, Slovenia: Slovenian Veterinary Research.

[CIT0018] Ellis, J. L., E. Kebreab, N. E. Odongo, B. W. McBride, E. K. Okine, and J. France. 2007. Prediction of methane production from dairy and beef cattle. J. Dairy Sci. 90:3456–3466. doi: https://doi.org/10.3168/jds.2006-675 17582129

[CIT0019] Ferreira, M. E., L. G. Ferreira, E. M. Latrubesse, and F. Miziara. 2016. Considerations about the land use and conversion trends in the savanna environments of Central Brazil under a geomorphological perspective. J Land Use Sci. 11:33–47. doi: https://doi.org/10.1080/1747423x.2013.845613

[CIT0020] Fraser, M. D., H. E. Vallin, and B. P. Roberts. 2022. Animal board invited review: Grassland-based livestock farming and biodiversity. Animal 16:100671. doi: https://doi.org/10.1016/j.animal.2022.100671 36436479 PMC9763128

[CIT0021] Fuller, K. W. 1967. Automated determination of sugars. In: Automation in analytical chemistry. Vol. II. European Technicon Symposium; p. 57–61.

[CIT0022] Hatew, B., S. C. Podesta, H. Van Laar, W. F. Pellikaan, J. L. Ellis, J. Dijkstra, and A. Bannink. 2015. Effects of dietary starch content and rate of fermentation on methane production in lactating dairy cows. J. Dairy Sci. 98:486–499. doi: https://doi.org/10.3168/jds.2014-8427 25465630

[CIT0023] Herrero, M., B. Henderson, P. Havlík, P. K. Thornton, R. T. Conant, P. Smith, S. Wirsenius, A. N. Hristov, P. Gerber, M. Gill, et al 2016. Greenhouse gas mitigation potentials in the livestock sector. Nat. Clim. Change 6:452–461. doi: https://doi.org/10.1038/nclimate2925

[CIT0024] Hoekstra, N. J., R. P. O. Schulte, P. C. Struik, and E. A. Lantinga. 2007. Pathways to improving the N efficiency of grazing bovines. Eur. J. Agron. 26:363–374. doi: https://doi.org/10.1016/j.eja.2006.12.002

[CIT0025] Huang, Q., X. Liu, G. Zhao, T. Hu, and Y. Wang. 2018. Potential and challenges of tannins as an alternative to in-feed antibiotics for farm animal production. Animal Nutri. (Zhongguo xu mu shou yi xue hui). 4:137–150. doi: https://doi.org/10.1016/j.aninu.2017.09.004 PMC6104569 30140753

[CIT0026] Johnson, K. A., and D. E. Johnson. 1995. Methane emissions from cattle. J. Anim. Sci. 73:2483–2492. doi: https://doi.org/10.2527/1995.7382483x 8567486

[CIT0027] Johnston, D. J., K. Theodoridou, and C. P. Ferris. 2019. The impact of field bean inclusion level in dairy cow diets on cow performance and nutrient utilisation. Livestock Sci. 220:166–172. doi: https://doi.org/10.1016/j.livsci.2018.12.015 31548054

[CIT0028] Keller, M., B. Reidy, A. Scheurer, L. Eggerschwiler, I. Morel, and K. Giller. 2021. Soybean meal can be replaced by faba beans, pumpkin seed cake, spirulina or be completely omitted in a forage-based diet for fattening bulls to achieve comparable performance, carcass and meat quality. Animals 11:1588. doi: https://doi.org/10.3390/ani11061588 34071418 PMC8227232

[CIT0029] Klopatek, S. C., E. Marvinney, T. Duarte, A. Kendall, X. C. Yang, and J. W. Oltjen. 2022. Grass-fed vs. grain-fed beef systems: performance, economic, and environmental trade-offs. J. Anim. Sci. 100:skab374. doi: https://doi.org/10.1093/jas/skab374 34936699 PMC8867585

[CIT0030] Laubach, J., F. M. Kelliher, T. W. Knight, H. Clark, G. Molano, and A. Cavanagh. 2008. Methane emissions from beef cattle a comparison of paddock- and animal-scale measurements. Aust. J. Exp. Agric. 48:132–137. doi: https://doi.org/10.1071/ea07256

[CIT0031] Llonch, P., S. M. Troy, C. -A. Duthie, M. Somarriba, J. Rooke, M. J. Haskell, R. Roehe, and S. P. Turner. 2018. Changes in feed intake during isolation stress in respiration chambers may impact methane emissions assessment. Animal Prod. Sci. 58:1011–1016. doi: https://doi.org/10.1071/an15563

[CIT0032] MacRae, J. C., and D. G. Armstrong. 1968. Enzyme method for determination of α-linked glucose polymers in biological materials. J. Sci. Food Agric. 19:578–581. doi: https://doi.org/10.1002/jsfa.2740191006

[CIT0033] Martin, C., C. Philippeau, and B. Michalet-Doreau. 1999. Effect of wheat and corn variety on fiber digestion in beef steers fed high-grain diets. J. Anim. Sci. 77:2269–2278. doi: https://doi.org/10.2527/1999.7782269x 10462008

[CIT0034] McDonald, P., R. A. Edwards, J. F. D. Greenhalgh, C. A. Morgan, L. A. Sinclair, and R. G. Wilkinson. 2010. Animal nutrition. 7th ed. Harlow, England: Prentice Hall/Pearson.

[CIT0035] Mertens, D. R. 2002. Gravimetric determination of amylase-treated neutral detergent fiber in feeds with refluxing in beakers or crucibles: collaborative study. J. AOAC Int. 85:1217–1240. doi: https://doi.org/10.1093/jaoac/85.6.1217 12477183

[CIT0036] Mertens, D. R. 2009. Impact of NDF content and digestibility on dairy cow performance. In: Proceedings of the 27th Annual Western Canadian Dairy Seminar. Advances in Dairy Technology, Red Deer, Alberta, University of Alberta, Edmonton, Canada; 21:191–201.

[CIT0037] Moate, P. J., S. R. O. Williams, C. Grainger, M. C. Hannah, E. N. Ponnampalam, and R. J. Eckard. 2011. Influence of cold-pressed canola, brewers grains and hominy meal as dietary supplements suitable for reducing enteric methane emissions from lactating dairy cows. Anim. Feed Sci. Technol. 166-167:254–264. doi: https://doi.org/10.1016/j.anifeedsci.2011.04.069

[CIT0038] Mussatto, S. I., G. Dragone, and I. C. Roberto. 2006. Brewers’ spent grain: generation, characteristics and potential applications. J. Cereal Sci. 43:1–14. doi: https://doi.org/10.1016/j.jcs.2005.06.001

[CIT0039] Owens, F. N., and B. A. Gardner. 2000. A review of the impact of feedlot management and nutrition on carcass measurements of feedlot cattle. J. Anim. Sci. 77:1–18. doi: https://doi.org/10.2527/jas2000.00218812007700es0034x

[CIT0040] Patra, A. K. 2013. The effect of dietary fats on methane emissions, and its other effects on digestibility, rumen fermentation and lactation performance in cattle: a meta-analysis. Livestock Sci. 155:244–254. doi: https://doi.org/10.1016/j.livsci.2013.05.023

[CIT0041] Pexas, G., B. Doherty, and I. Kyriazakis. 2023. The future of protein sources in livestock feeds: implications for sustainability and food safety. Front Sust. Food Syst. 7:1188467. doi: https://doi.org/10.3389/fsufs.2023.1188467

[CIT0042] Pinheiro Machado Filho, L. C., H. L. S. Seó, R. R. Daros, D. Enriquez-Hidalgo, A. V. Wendling, and L. C. Pinheiro Machado. 2021. Voisin rational grazing as a sustainable alternative for livestock production. Animals 11:3494. doi: https://doi.org/10.3390/ani11123494 34944271 PMC8698051

[CIT0043] Poore, M. H., J. A. Moore, T. P. Eck, and C. B. Theurer. 1993. Effect of fiber source and ruminal starch degradability on site and extent of digestion in dairy cows. J. Dairy Sci. 76:2244–2253. doi: https://doi.org/10.3168/jds.s0022-0302(93)77561-x

[CIT0044] Puhakka, L., S. Jaakkola, I. Simpura, T. Kokkonen, and A. Vanhatalo. 2016. Effects of replacing rapeseed meal with fava bean at 2 concentrate crude protein levels on feed intake, nutrient digestion, and milk production in cows fed grass silage–based diets. J. Dairy Sci. 99:7993–8006. doi: https://doi.org/10.3168/jds.2016-10925 27522411

[CIT0045] Ramin, M., and P. Huhtanen. 2013. Development of equations for predicting methane emissions from ruminants. J. Dairy Sci. 96:2476–2493. doi: https://doi.org/10.3168/jds.2012-6095 23403199

[CIT0046] Ribas-Agustí, A., I. Díaz, C. Sárraga, J. A. García-Regueiro, and M. Castellari. 2019. Nutritional properties of organic and conventional beef meat at retail. J. Sci. Food Agric. 99:4218–4225. doi: https://doi.org/10.1002/jsfa.9652 30790287

[CIT0047] Roberston, J. B., and P. J. Van Soest. 1981. The detergent system of analysis and its application to human foods. In: James, W. and O. Theander, editors. The analysis of dietary fiber in food no. 3. New York (NY): Marcel Dekker Inc.; p. 123–158.

[CIT0048] Salah, N., D. Sauvant, and H. Archimede. 2015. Response of growing ruminants to diet in warm climates: a meta-analysis. Animal 9:822–830. doi: https://doi.org/10.1017/S175173111400322X 25602578

[CIT0049] Santos-Silva, J., S. P. Alves, A. Francisco, A. P. Portugal, M. T. Dentinho, J. Almeida, J. L. R. da Silva, L. Fialho, L. Cachucho, E. Jerónimo, et al 2023. Forage based diet as an alternative to a high concentrate diet for finishing young bulls - effects on growth performance, greenhouse gas emissions and meat quality. Meat Sci. 198:109098. doi: https://doi.org/10.1016/j.meatsci.2023.109098 36681060

[CIT0050] Smith, D., G. M. Paulsen, and C. A. Raguse. 1964. Extraction of total available carbohydrates from grass and legume tissue. Plant Physiol. 39:960–962. doi: https://doi.org/10.1104/pp.39.6.960 16656042 PMC550200

[CIT0051] Song, X. -P., M. C. Hansen, P. Potapov, B. Adusei, J. Pickering, M. Adami, A. Lima, V. Zalles, S. V. Stehman, C. M. Di Bella, et al 2021. Massive soybean expansion in South America since 2000 and implications for conservation. Nat. Sustainability 4:784–792. doi: https://doi.org/10.1038/s41893-021-00729-z PMC8350977 34377840

[CIT0052] Soxhlet, F. 1879. Die gewichtsanalytische Bestimmung des Milchfettes. Dinglers Polytechnisches J. 232:461–465.

[CIT0053] Średnicka-Tober, D., M. Barański, C. Seal, R. Sanderson, C. Benbrook, H. Steinshamn, J. Gromadzka-Ostrowska, E. Rembiałkowska, K. Skwarło-Sońta, M. Eyre, et al 2016. Composition differences between organic and conventional meat: a systematic literature review and meta-analysis. Br. J. Nutr. 115:994–1011. doi: https://doi.org/10.1017/S0007114515005073 26878675 PMC4838835

[CIT0054] Tas, B. M., H. Z. Taweel, H. J. Smit, A. Elgersma, J. Dijkstra, and S. Tamminga. 2006. Effects of perennial ryegrass cultivars on milk yield and nitrogen utilization in grazing dairy cows. J. Dairy Sci. 89:3494–3500. doi: https://doi.org/10.3168/jds.S0022-0302(06)72388-8 16899684

[CIT0055] Thompson, L. R., M. R. Beck, S. A. Gunter, G. D. Williams, S. E. Place, and R. R. Reuter. 2019. An energy and monensin supplement reduces methane emission intensity of stocker cattle grazing winter wheat. Appl. Ani. Sci. 35:433–440. doi: https://doi.org/10.15232/aas.2018-01841

[CIT0056] Van Lingen, H. J., M. Niu, E. Kebreab, S. C. Valadares Filho, J. A. Rooke, C. -A. Duthie, A. Schwarm, M. Kreuzer, P. I. Hynd, M. Caetano, et al 2019. Prediction of enteric methane production, yield and intensity of beef cattle using an intercontinental database. Agri, Ecosyst Environ. 283:106575. doi: https://doi.org/10.1016/j.agee.2019.106575

[CIT0057] Wägeli, S., M. Janssen, and U. Hamm. 2015. Organic consumers’ preferences and willingness‐to‐pay for locally produced animal products. Int. J. Consum. Stud. 40:357–367. doi: https://doi.org/10.1111/ijcs.12262

[CIT0058] Waghorn, G. C., and D. A. Clark. 2004. Feeding value of pastures for ruminants. N. Z. Vet. J. 52:320–331. doi: https://doi.org/10.1080/00480169.2004.36448 15768132

[CIT0059] Weiss, W. P., D. G. Chamberlain, and C. W. Hunt. 2003. Feeding silages. In: Buxton, D. R., R. E. Muck, and J. H. Harrison, editors. Silage science and technology. Madison: American Society of Agronomy; p. 469–504.

[CIT0060] Yan, T., J. P. Frost, T. W. J. Keady, R. E. Agnew, and C. S. Mayne. 2007. Prediction of nitrogen excretion in feces and urine of beef cattle offered diets containing grass silage1. J. Anim. Sci. 85:1982–1989. doi: https://doi.org/10.2527/jas.2006-408 17504962

[CIT0061] Yan, T., M. G. Porter, and C. S. Mayne. 2009. Prediction of methane emission from beef cattle using data measured in indirect open-circuit respiration calorimeters. Animal 3:1455–1462. doi: https://doi.org/10.1017/S175173110900473X 22444941

[CIT0062] Zeko-Pivac, A., M. Tisma, P. Znidarsic-Plazl, B. Kulisic, G. Sakellaris, J. Hao, and M. Planinic. 2022. The potential of brewer’s spent grain in the circular bioeconomy: state of the art and future perspectives. Front. Bioeng. Biotechnol. 10:870744. doi: https://doi.org/10.3389/fbioe.2022.870744 35782493 PMC9247607

