# Supplementary material for: Nutrient use and methane emissions in growing beef fed different protein sources and a pasture-based diet
Source: J Anim Sci. 2025 Jan 17;103:skaf007. doi: 10.1093/jas/skaf007 (PMC11815277; doi:10.1093/jas/skaf007)
Supplement: skaf007_suppl_Supplementary_Material [file skaf007_suppl_supplementary_material.docx]

**Nutrient use and methane emissions in growing beef fed different protein sources and a pasture-based diet**

Christos Christodoulou ^†^, Kirsty E. Kliem ^†^, Marc D. Auffret ^‡^, David J. Humphries ^§^, Paul Kirton ^§^, Hassan Jalal ^#^, John R. Newbold ^||^, Nicholas Davison ^†^, Laurence G. Smith ^†, ¶^, Sokratis Stergiadis ^†, 1^

^†^ *University of Reading, School of Agriculture, Policy and Development, Earley gate, RG6 6EU Reading, United Kingdom*

*^‡^ Agrifirm, Booiebos 5, B-9031 Gent (Drongen), Belgium*

*^§^ Centre for Dairy Research, School of Agriculture, Policy and Development, University of Reading, Hall Farm House, Church Ln, Reading RG2 9HX, United Kingdom*

*^#^ Department of Veterinary Medicine, University of Teramo, Piano d’Accio, Teramo, 64100, Italy*

*^||^ Scotland's Rural College, Dairy Research & Innovation Centre, Barony Campus, Parkgate, DG1 3NE Dumfries, United Kingdom*

*^¶^ Swedish University of Agricultural Sciences, Biosystem & teknologi, SE-234 22 Lomma, Sweden*

*^1^* Corresponding author: Email address: [s.stergiadis@reading.ac.uk](mailto:s.stergiadis@reading.ac.uk) (Sokratis Stergiadis), full postal address: Department of Animal Sciences, School of Agriculture, Policy, and Development, University of Reading, Earley Gate, PO Box 237, RG6 6EU, Reading, United Kingdom

Journal of Animal Science

| **Table S1.** Feed and nutrient intakes (kg/day), growth measurements, and enteric CH_4_ emissions from growing beef (heifers, steers) fed the experimental diets during the group-housed period of the animal trial | | | | | | | | | | | | | | |
| --- | --- | --- | --- | --- | --- | --- | --- | --- | --- | --- | --- | --- | --- | --- |
| Item^3^ | Dietary treatments (D)^1^ | | |  | Sex (S) | |  | *P*-values^2^ | | | | | | |
|  | SB | BSG | BNS | SEM | Heifers | Steers | SEM | D | Week (W) | (S) | D × W | D × S | W × S | D × W × S |
| **Feed and nutrient intakes (kg/day)** | | | | | | | | | | | | | | |
| DM^4^ | 9.78^a^ | 8.51^b^ | 9.73^a^ | 0.277 | 8.55 | 10.14 | 0.227 | 0.007 | <0.001 | <0.001 | 0.926 | 0.987 | 0.879 | 0.985 |
| OM^5^ | 9.44^a^ | 8.29^b^ | 9.58^a^ | 0.272 | 8.26 | 9.95 | 0.222 | 0.006 | <0.001 | <0.001 | 0.847 | 0.878 | 0.995 | 0.995 |
| GE intake (MJ/day)^5^ | 168 | 157 | 170 | 4.84 | 150 | 180 | 3.95 | 0.143 | 0.031 | <0.001 | 0.656 | 0.815 | 0.998 | 0.995 |
| NI | 0.20 | 0.18 | 0.19 | 0.006 | 0.17 | 0.21 | 0.005 | 0.084 | <0.001 | <0.001 | 0.151 | 0.831 | 0.999 | 0.999 |
| NDF^5^ | 4.16 | 4.04 | 3.94 | 0.119 | 3.67 | 4.43 | 0.097 | 0.437 | <0.001 | <0.001 | 0.135 | 0.702 | 0.998 | 0.997 |
| ADF^5^ | 2.60 | 2.36 | 2.37 | 0.074 | 2.22 | 2.67 | 0.060 | 0.053 | <0.001 | <0.001 | 0.045 | 0.804 | 0.998 | 0.998 |
| Oil^5^ | 0.32^b^ | 0.43^a^ | 0.31^b^ | 0.010 | 0.32 | 0.39 | 0.008 | <0.001 | <0.001 | <0.001 | 0.078 | 0.206 | 0.999 | 0.998 |
| EE^5^ | 0.24^b^ | 0.35^a^ | 0.23^b^ | 0.008 | 0.25 | 0.30 | 0.006 | <0.001 | <0.001 | <0.001 | 0.101 | 0.131 | 0.999 | 0.998 |
| Starch^5^ | 2.07^b^ | 1.72^c^ | 2.58^a^ | 0.064 | 1.93 | 2.32 | 0.052 | <0.001 | <0.001 | <0.001 | <0.001 | 0.697 | 0.999 | 0.993 |
| WSC^5^ | 0.28^a^ | 0.16^c^ | 0.24^b^ | 0.007 | 0.21 | 0.25 | 0.006 | <0.001 | <0.001 | <0.001 | 0.027 | 0.869 | 0.995 | 1.000 |
| **Growth measurements** | | | | | | | | | | | | | | |
| BW (kg)^6^ | 469 | 464 | 477 | 11.5 | 472 | 475 | 9.4 | 0.943 | <0.001 | 0.784 | 0.996 | 0.381 | 0.580 | 0.886 |
| BWc (kg/day)^6^ | 1.42 | 1.36 | 1.57 | 0.122 | 0.94 | 1.96 | 0.099 | 0.598 | <0.001 | <0.001 | 0.992 | 0.853 | 0.615 | 0.694 |
| BWc/DMI (kg/kg)^6^ | 0.14 | 0.16 | 0.15 | 0.01 | 0.12 | 0.18 | 0.01 | 0.606 | <0.001 | 0.001 | 0.722 | 0.924 | 0.058 | 0.191 |
| **Enteric CH_4_ emissions** | | | | | | | | | | | | | | |
| CH_4_ production (g/day)^6^ | 230 | 213 | 216 | 8.7 | 214 | 226 | 7.1 | 0.346 | 0.012 | 0.232 | 0.135 | 0.202 | 0.372 | 0.654 |
| CH_4_/DMI (g/kg) | 24.2^ab^ | 26.4^a^ | 22.5^b^ | 0.98 | 25.6 | 23.1 | 0.75 | 0.028 | <0.001 | 0.034 | 0.648 | 0.066 | 0.642 | 0.967 |
| CH_4_/OMI (g/kg)^5^ | 25.4^ab^ | 27.5^a^ | 23.4^b^ | 0.99 | 26.9 | 23.9 | 0.81 | 0.016 | <0.001 | 0.009 | 0.438 | 0.025 | 0.981 | 0.992 |
| CH_4_/BW (g/kg)^6^ | 0.50 | 0.44 | 0.46 | 0.023 | 0.45 | 0.48 | 0.019 | 0.292 | <0.001 | 0.196 | 0.177 | 0.422 | 0.296 | 0.673 |
| CH_4_/BWg (g/kg)^8^ | 257 | 205 | 248 | 30.2 | 286 | 192 | 24.7 | 0.400 | <0.001 | 0.007 | 0.466 | 0.169 | 0.316 | 0.270 |
| CH_4_-E/GEI (MJ/MJ)^5^ | 0.08 | 0.08 | 0.07 | 0.003 | 0.08 | 0.07 | 0.002 | 0.115 | 0.054 | 0.007 | 0.290 | 0.016 | 0.903 | 0.949 |
| ^1^ SB = Total mixed ratio (TMR) including soy as the main protein source; BSG = TMR including local brewers’ spent grains as the main protein source; BNS = TMR including local field beans as the main protein source.  ^2^ Significances were declared at *P*<0.05. Significant differences between dietary treatments within variables are indicated with different superscript letters according to Fisher's Least Significant Difference (LSD) test.  ^3^ DM = dry matter; OM = organic matter; GE = gross energy; N= Nitrogen; ADF = acid detergent fiber; NDF = neutral detergent fiber; EE = Ether extract; WSC = Water soluble carbohydrates; BW = body weight; BWg = body weight gain; CH_4_ = methane; DMI = DM intake; OMI = OM intake; CH_4_-E = CH_4_ energy output; GEI = GE intake.  ^4^ measurements for these variables were n=144 for SB, n=144 for BSG, and n=144 for BNS.  ^5^ measurements for these variables were n=95 for SB, n=95 for BSG, and n=96 for BNS.  ^6^ measurements for this variable were n=104 for SB, n=104 for BSG, and n=104 for BNS.  ^7^ measurements for this variable were n=111 for SB, n=99 for BSG, and n=108 for BNS.  ^8^ for weeks where an animal has lost weight (BW change ≤ 0) the corresponding measurement was excluded from the dataset. Therefore, the number of measurements per treatment were n=66 for SB, n=65 for BSG, and n=72 for BNS. | | | | | | | | | | | | | | |

| **Table S2.** Feed and nutrient intakes (kg/day) and digestibility (kg/kg) from steers fed the experimental diets during the chamber measurement periods of the animal trial. | | | | | | | | | | | | | | |
| --- | --- | --- | --- | --- | --- | --- | --- | --- | --- | --- | --- | --- | --- | --- |
|  | Dietary treatments (D)^1^ | | | | | | SEM | | *P*-values^2^ | | | | | |
| Item^3^ | SB  (n=16) | | BSG  (n=16) | | BNS  (n=15^4^) | |  |  | D | | Period (P) | | D × P | |
| **Feed and nutrient intakes (kg/day)** | | | | | | | | | | | | | | |
| DM | 5.71 | | 5.84 | | 5.67 | | 0.350 | | 0.932 | | <0.001 | | 0.725 | |
| OM | 5.41 | | 5.56 | | 5.36 | | 0.313 | | 0.900 | | <0.001 | | 0.769 | |
| GE | 96.3 | | 105 | | 96.3 | | 5.70 | | 0.475 | | 0.006 | | 0.908 | |
| N | 113 | | 120 | | 104 | | 6.6 | | 0.309 | | 0.014 | | 0.762 | |
| NDF | 2.38 | | 2.71 | | 2.21 | | 0.152 | | 0.142 | | <0.001 | | 0.375 | |
| ADF | 1.49 | | 1.59 | | 1.31 | | 0.104 | | 0.233 | | 0.003 | | 0.383 | |
| Oil | 0.18^b^ | | 0.29^a^ | | 0.17^b^ | | 0.013 | | <0.001 | | 0.005 | | 0.509 | |
| EE | 0.14^b^ | | 0.23^a^ | | 0.13^b^ | | 0.010 | | <0.001 | | 0.007 | | 0.831 | |
| Starch | 1.18^b^ | | 1.15^b^ | | 1.46^a^ | | 0.072 | | 0.035 | | 0.007 | | 0.014 | |
| WSC | 0.17^a^ | | 0.11^b^ | | 0.14^ab^ | | 0.010 | | 0.016 | | 0.018 | | 0.808 | |
| **Digestibility (kg/kg)** |  | |  | |  | |  | |  | |  | |  | |
| DM | 0.70^a^ | | 0.66^b^ | | 0.68^ab^ | | 0.007 | | 0.004 | | 0.347 | | 0.201 | |
| OM | 0.73^b^ | | 0.69^c^ | | 0.71^a^ | | 0.005 | | 0.004 | | 0.225 | | 0.311 | |
| DOMD | 0.69^a^ | | 0.65^b^ | | 0.67^ab^ | | 0.006 | | 0.006 | | 0.387 | | 0.238 | |
| N | 0.59^ab^ | | 0.61^a^ | | 0.54^b^ | | 0.011 | | 0.010 | | 0.040 | | 0.411 | |
| NDF | 0.64^a^ | | 0.59^b^ | | 0.57^b^ | | 0.008 | | 0.001 | | 0.585 | | 0.682 | |
| ADF | 0.57^a^ | | 0.52^b^ | | 0.47^b^ | | 0.014 | | 0.007 | | 0.498 | | 0.649 | |
| ^1^ SB = Total mixed ratio (TMR) including soy as the main protein source; BSG = TMR including local brewers’ spent grains as the main protein source; BNS = TMR including local field beans as the main protein source.  ^2^ Significances were declared at *P*<0.05. Significant differences between dietary treatments within variable are indicated with different superscript letters according to Fisher's Least Significant Difference (LSD) test.  ^3^ DM = dry matter; OM = organic matter; N = nitrogen; NDF = neutral detergent fiber; ADF = acid detergent fiber; EE = ether extract; WSC = water soluble carbohydrates; DOMD = digestible OM in DM; GE = gross energy.  ^4^ there was a missing measurement in one animal in the BNS treatment in the last period of the experiment. | | | | | | | | | | | | | | |
| **Table S3.** Energy metabolism and enteric CH_4_ emissions from steers fed the experimental diets during the chamber measurement periods of the animal trial. | | | | | | | | | | | | | | |
| Item^3^ | | Dietary treatments (D)^1^ | | | | | | SEM | | *P*-values^2^ | | | | |
|  |  | SB  (n=16) | | BSG  (n=16) | | BNS  (n=15^4^) | |  |  | D | | Period (P) | | D × P |
| **Energy intakes and outputs (MJ/day)** | | | | | | | | | | | | | | |
| GE intake | | 96.3 | | 105 | | 96.3 | | 5.70 | | 0.475 | | 0.006 | | 0.908 |
| Fecal E output | | 31.5 | | 38.1 | | 33.8 | | 1.84 | | 0.109 | | <0.001 | | 0.165 |
| Urine E output | | 2.26 | | 2.76 | | 2.12 | | 0.259 | | 0.263 | | 0.235 | | 0.055 |
| CH_4_-E output | | 13.4 | | 13.0 | | 9.92 | | 1.926 | | 0.426 | | 0.002 | | 0.829 |
| DE intake | | 64.8 | | 67.1 | | 62.9 | | 4.35 | | 0.792 | | 0.028 | | 0.776 |
| ME intake | | 61.8 | | 63.7 | | 60.1 | | 4.22 | | 0.836 | | 0.034 | | 0.723 |
| **Energy utilisation (MJ/MJ)** | | | | | | | | | | | | | | |
| DE/GE | | 0.67 | | 0.64 | | 0.65 | | 0.010 | | 0.098 | | 0.393 | | 0.218 |
| ME/GE | | 0.64 | | 0.61 | | 0.62 | | 0.011 | | 0.148 | | 0.555 | | 0.116 |
| ME/DE | | 0.95 | | 0.95 | | 0.96 | | 0.004 | | 0.600 | | 0.310 | | 0.052 |
| Fecal E output/GEI | | 0.33 | | 0.36 | | 0.35 | | 0.010 | | 0.098 | | 0.393 | | 0.218 |
| Urine E output/GEI | | 0.024 | | 0.026 | | 0.022 | | 0.003 | | 0.522 | | 0.169 | | 0.026 |
| **Enteric CH_4_ emissions** | | | | | | | | | | | | | | |
| CH_4_ production (g/day) | | 241 | | 234 | | 230 | | 8.3 | | 0.639 | | <0.001 | | 0.442 |
| CH_4_/DMI (g/kg) | | 42.7 | | 40.1 | | 41.0 | | 1.32 | | 0.406 | | 0.167 | | 0.383 |
| CH_4_/OMI (g/kg) | | 45.1 | | 42.1 | | 46.8 | | 1.55 | | 0.179 | | 0.219 | | 0.407 |
| CH_4_/digestible DMI (g/kg) | | 60.9 | | 61.2 | | 60.3 | | 2.35 | | 0.965 | | 0.164 | | 0.298 |
| CH_4_/digestible OMI (g/kg) | | 62.1 | | 61.6 | | 61.1 | | 2.38 | | 0.950 | | 0.195 | | 0.302 |
| CH_4_/BW (g/kg) | | 0.50 | | 0.51 | | 0.47 | | 0.018 | | 0.219 | | 0.057 | | 0.226 |
| CH_4_-E/GEI (MJ/MJ) | | 0.14 | | 0.12 | | 0.13 | | 0.005 | | 0.106 | | 0.632 | | 0.877 |
| CH_4_-E/DEI (MJ/MJ) | | 0.21 | | 0.20 | | 0.21 | | 0.010 | | 0.515 | | 0.517 | | 0.649 |
| CH_4_-E/MEI (MJ/MJ) | | 0.22 | | 0.21 | | 0.22 | | 0.011 | | 0.570 | | 0.650 | | 0.583 |
| **Other respirometry measurements** | | | | | | | | | | | | | | |
| CO_2_ production (L/day) | | 4012 | | 4103 | | 4183 | | 124.8 | | 0.635 | | 0.012 | | 0.028 |
| O_2_ production (L/day) | | 4129 | | 4180 | | 4148 | | 103.7 | | 0.940 | | 0.001 | | 0.019 |
| Heat production (MJ/day) | | 85.7 | | 86.3 | | 86.3 | | 2.08 | | 0.972 | | 0.003 | | 0.025 |
| Respiratory Quotient (L/day) | | 1.00 | | 0.98 | | 1.01 | | 0.007 | | 0.073 | | 0.983 | | 0.874 |
| ^1^ SB = Total mixed ratio (TMR) including soy as the main protein source; BSG = TMR including local brewers’ spent grains as the main protein source; BNS = TMR including local field beans as the main protein source.  ^2^ Significances were declared at *P*<0.05. Significant differences between dietary treatments within variable are indicated with different superscript letters according to Fisher's Least Significant Difference (LSD) test.  ^3^ GE = gross energy; E = energy; CH_4_ = methane; DE = digestible energy; ME = metabolisable energy; GEI = GE intake; DMI = dry matter intake; OMI = organic matter intake; BW = body weight; DEI = DE intake; MEI = ME intake.  ^4^ there was a missing measurement in one animal in the BNS treatment in the last period of the experiment. | | | | | | | | | | | | | | |

| **Table S4.** Nitrogen intake and outputs (g/day) and nitrogen utilisation (g/g) from steers fed the experimental diets during the chamber measurement periods of the animal trial. | | | | | | | |  |
| --- | --- | --- | --- | --- | --- | --- | --- | --- |
| Item^3^ | Dietary treatments (D)^1^ | | | SEM | *P*-values^2^ | | | |
|  | SB  (n=16) | BSG  (n=16) | BNS  (n=15^4^) |  | D | Period (P) | D × P | |
| **N intakes and outputs (g/day)** | | | | | | | |  |
| N intake | 113 | 120 | 104 | 6.6 | 0.309 | 0.014 | 0.762 | |
| Feces N output | 45.8 | 46.9 | 48.0 | 2.93 | 0.874 | <0.001 | 0.169 | |
| Urine N output | 57.8 | 66.8 | 45.2 | 5.19 | 0.071 | 0.074 | 0.446 | |
| Manure N output | 104^b^ | 114^a^ | 92.9^c^ | 2.42 | <0.001 | 0.014 | 0.171 | |
| Retained N | 9.58 | 6.21 | 9.75 | 9.913 | 0.961 | 0.973 | 0.394 | |
| **N utilisation (kg/kg)** | | | | | | | |  |
| Feces N output/NI | 0.41^ab^ | 0.39^b^ | 0.46^a^ | 0.011 | 0.009 | 0.045 | 0.423 | |
| Urine N output/ NI | 0.52 | 0.56 | 0.45 | 0.092 | 0.746 | 0.726 | 0.393 | |
| Manure N output/ NI | 0.93 | 0.95 | 0.92 | 0.075 | 0.936 | 0.974 | 0.401 | |
| Retained N/NI | 0.53 | 0.30 | 0.45 | 0.473 | 0.937 | 0.971 | 0.420 | |
| FNO/MNO | 0.45 | 0.42 | 0.54 | 0.044 | 0.193 | 0.177 | 0.113 | |
| UNO/MNO | 0.55 | 0.59 | 0.46 | 0.044 | 0.193 | 0.177 | 0.113 | |
| UNO/FNO | 1.29 | 1.43 | 1.01 | 0.185 | 0.294 | 0.167 | 0.308 | |
| ^1^ SB = Total mixed ratio (TMR) including soy as the main protein source; BSG = TMR including local brewers’ spent grains as the main protein source; BNS = TMR including local field beans as the main protein source.  ^2^ Significances were declared at *P*<0.05. Significant differences between dietary treatments within variable are indicated with different superscript letters according to Fisher's Least Significant Difference (LSD) test.  ^3^ N = nitrogen, NI = N intake; FNO = feces N output; MNO = manure N output; UNO = urine N output.  ^4^ there was a missing measurement in one animal in the BNS treatment in the last period of the experiment. | | | | | | | |  |

| 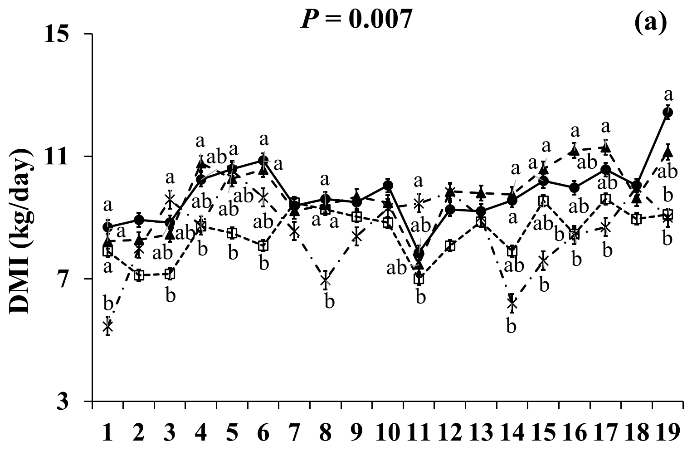 | 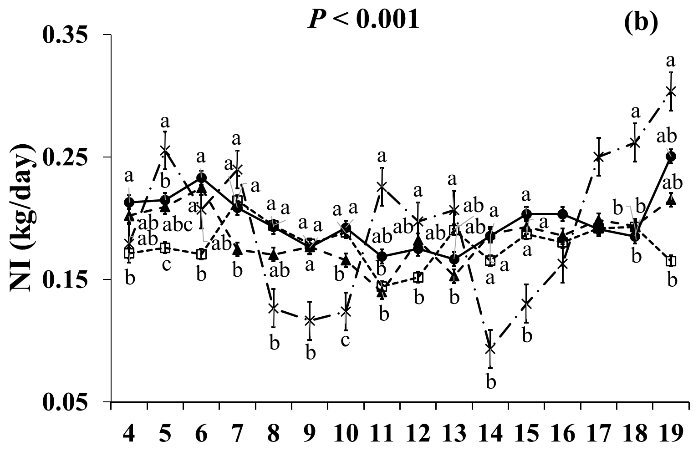 |
| --- | --- |
| 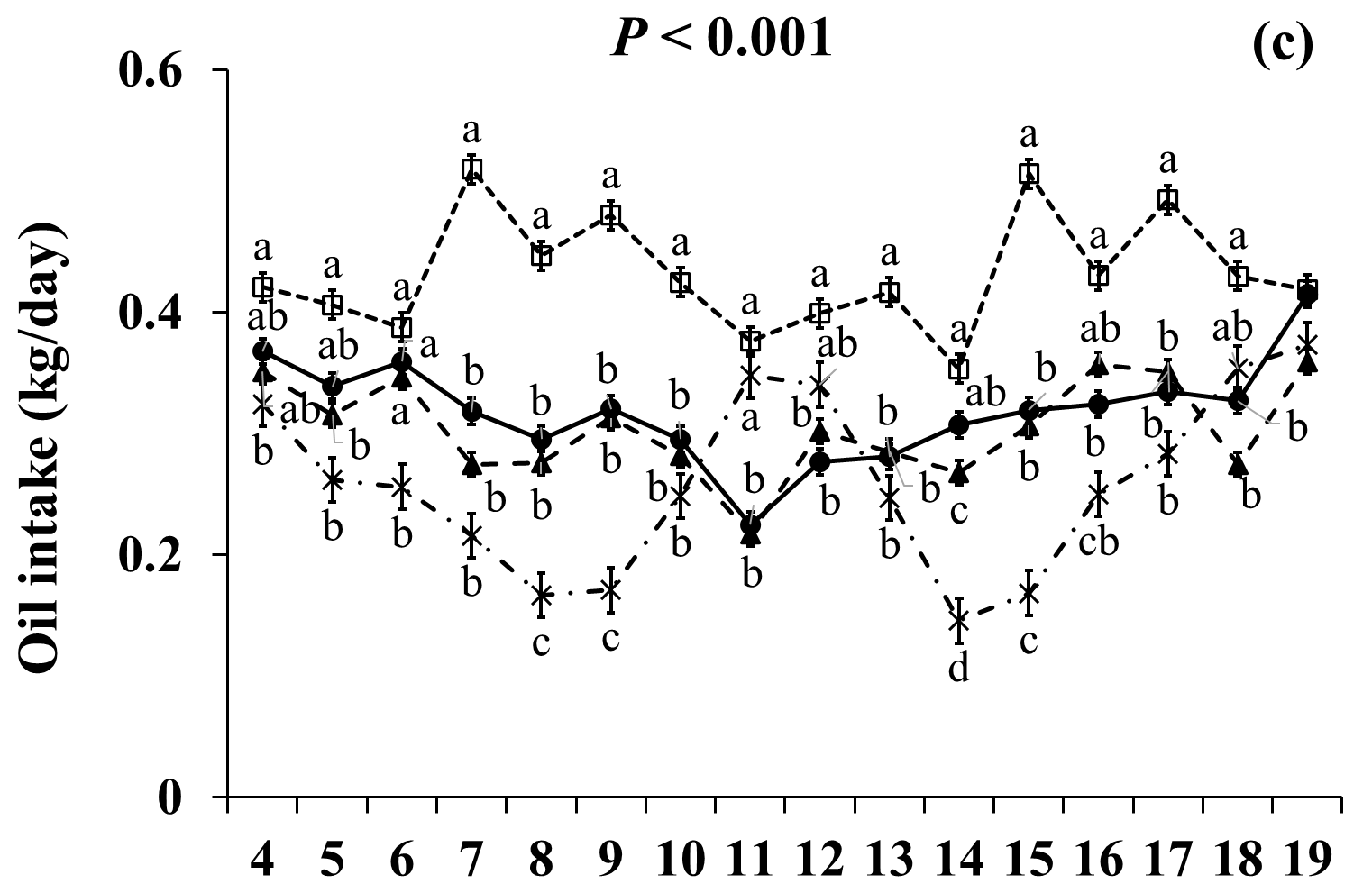 | 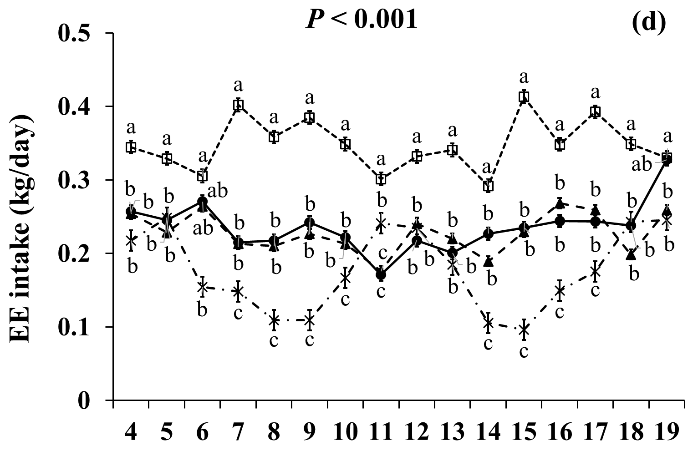 |
| 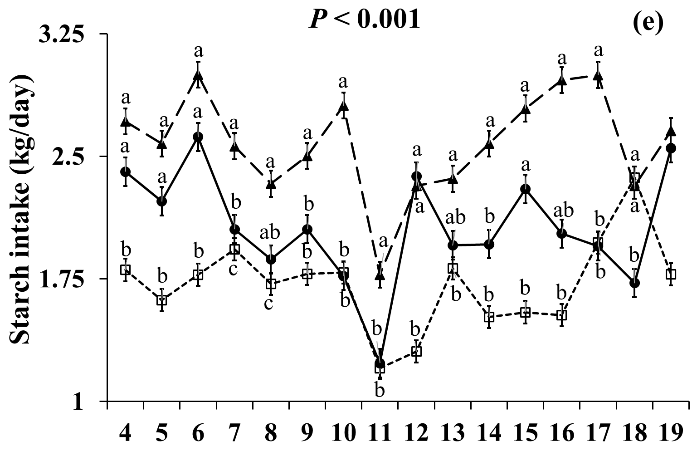 | 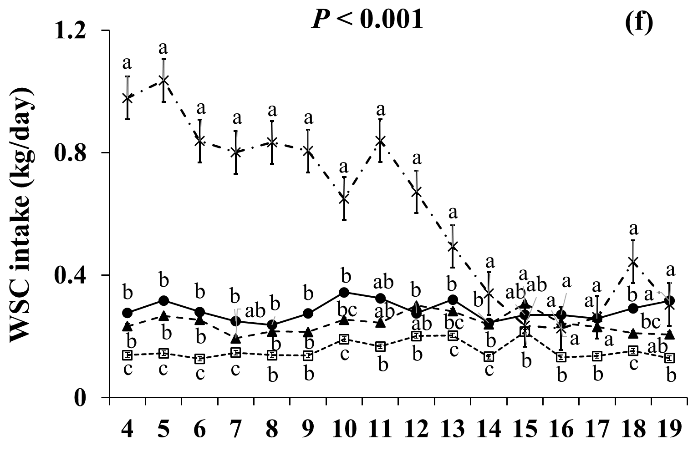 |
| 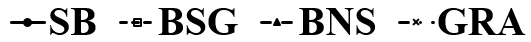 | |
| **Figure S1.** Significant effect of Treatment×Week interaction on dry matter intake (DMI, panel a), nitrogen intake (NI, panel b), oil intake (panel c), ether extract intake (EE, panel d), starch intake (panel e), and water-soluble carbohydrates (WSC) intake (panel f) in growing beef (heifers, steers) fed the experimental diets during the group-housed period of the animal trial. Significances were declared at *P* < 0.05. Significant differences within weeks are indicated with different superscript letters according to Fisher's Least Significant Difference (LSD) test (*P* < 0.05). Error bars represent standard error of means (SEM). | |

| 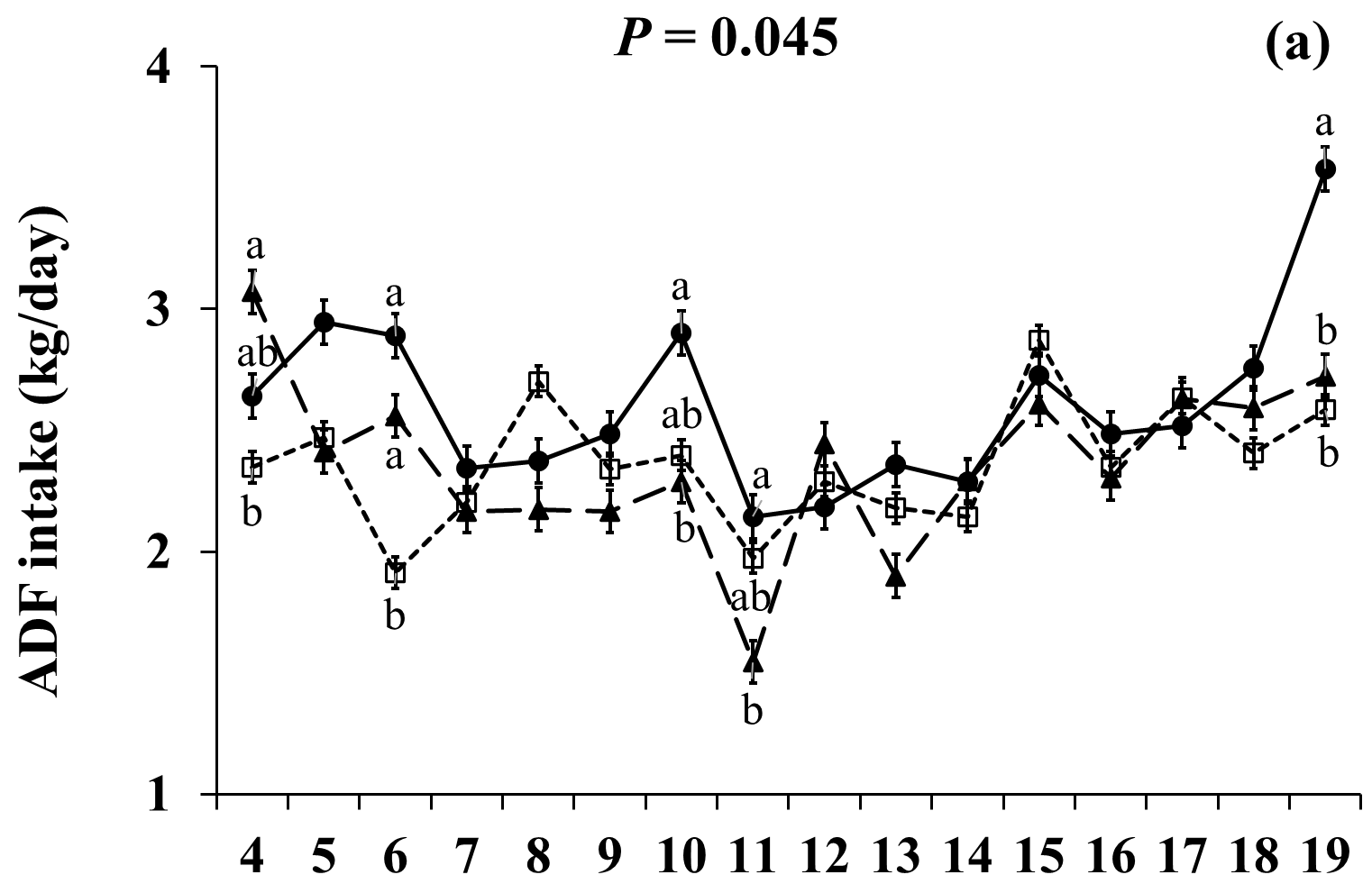 | 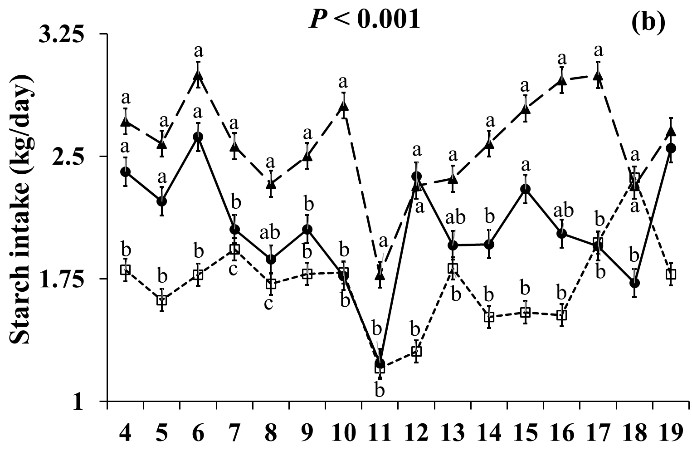 |
| --- | --- |
| 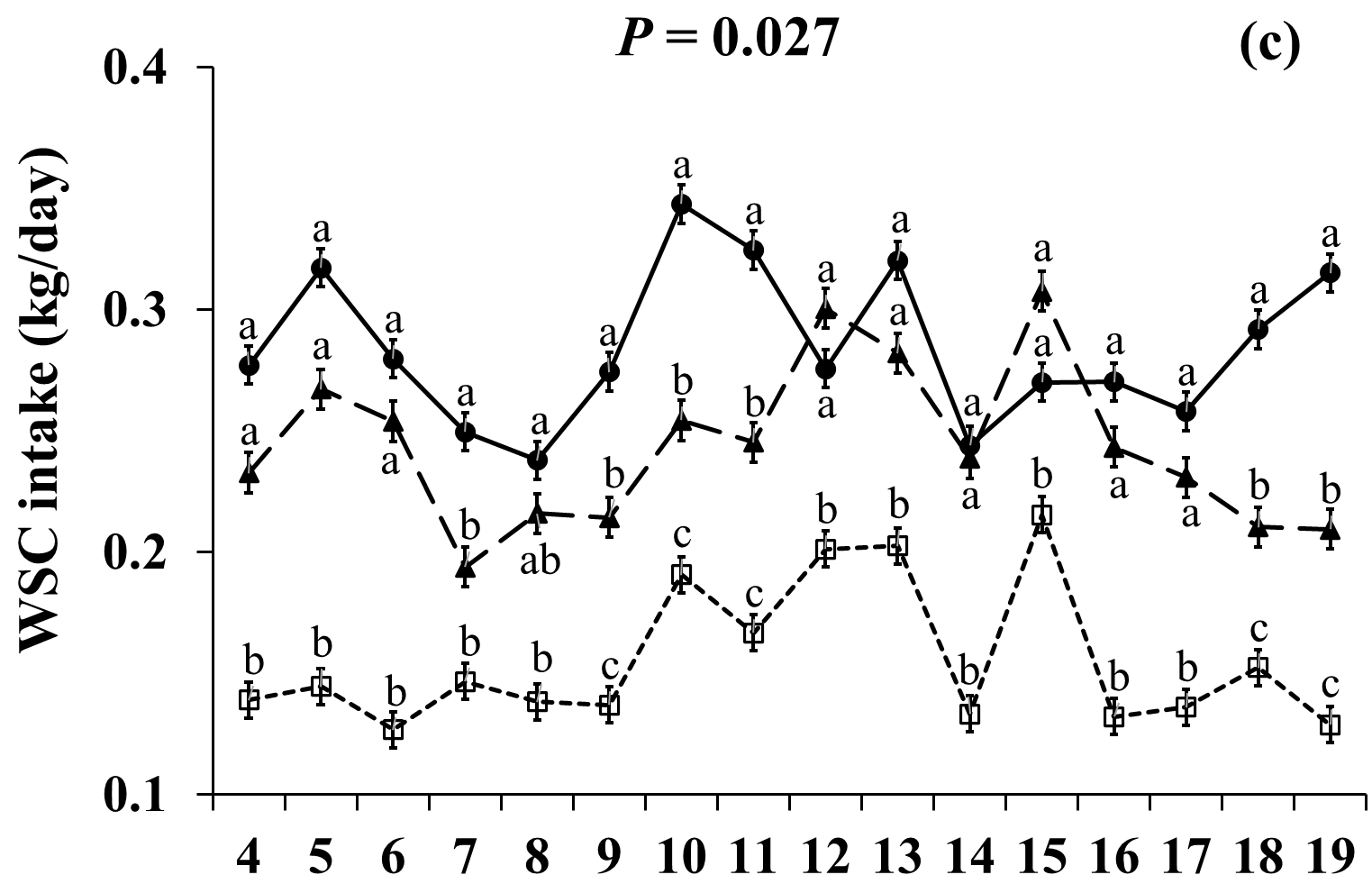 | 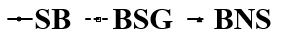  **Figure S2.** Significant effect of Treatment×Week interaction on acid detergent fiber (ADF) intake (panel a), starch intake (panel b), and water-soluble carbohydrates (WSC) intake (panel c), in growing beef (heifers, steers) fed the experimental diets during the group-housed period of the animal trial. Significances were declared at *P* < 0.05. Significant differences within weeks are indicated with different superscript letters according to Fisher's Least Significant Difference (LSD) test (*P* < 0.05). Error bars represent standard error of means (SEM). |

| 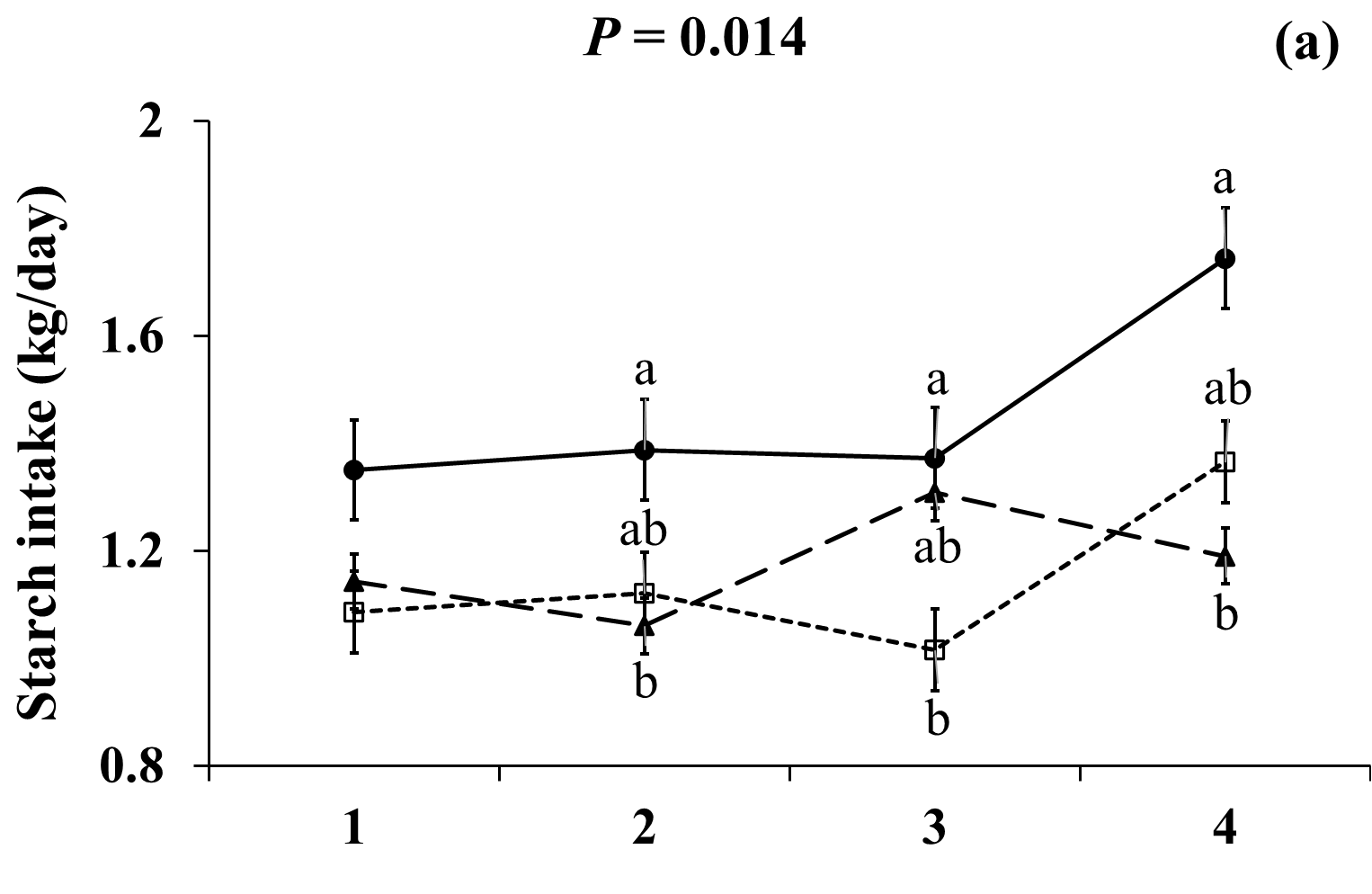 | 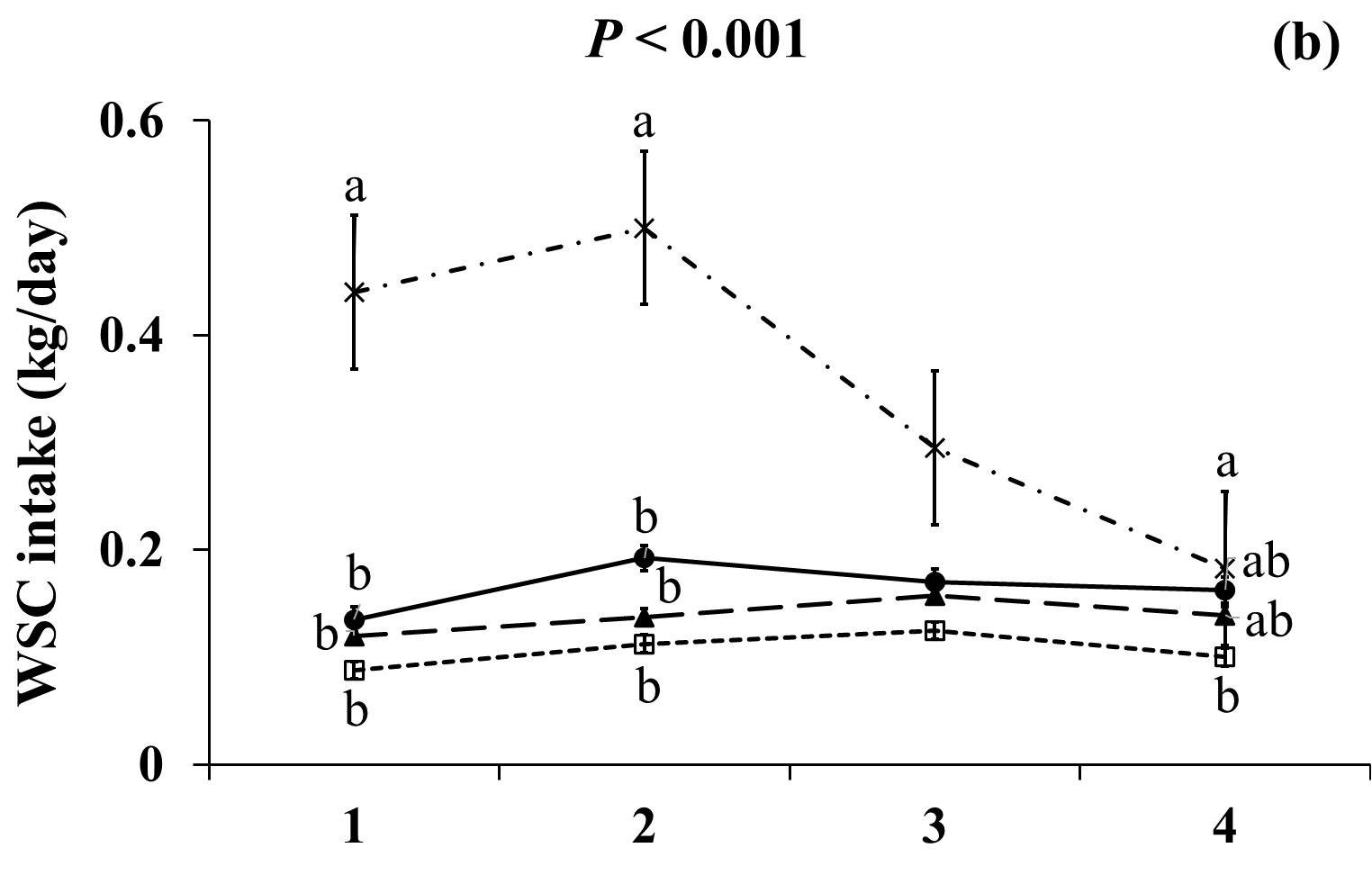 |
| --- | --- |
| 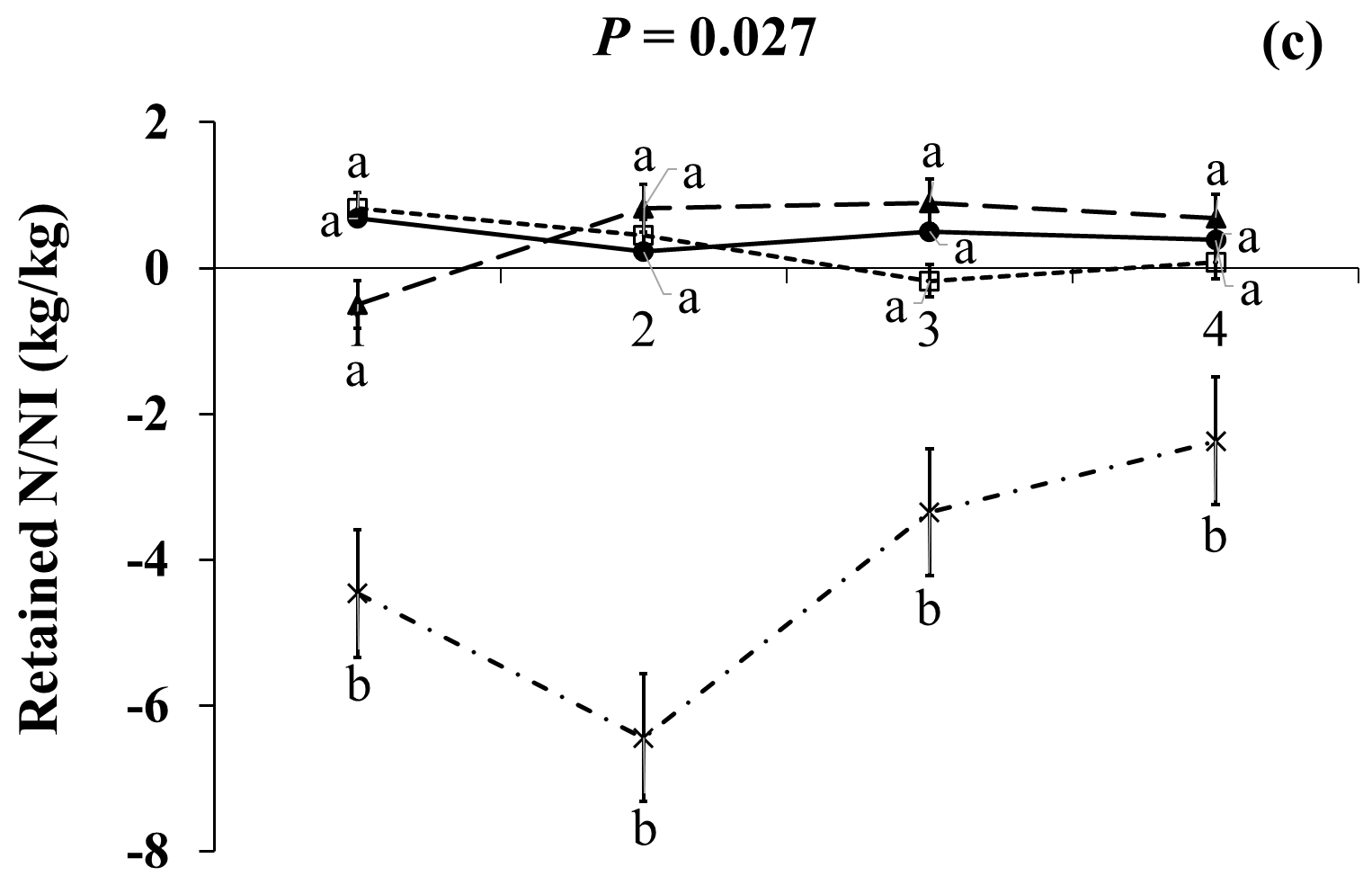 | 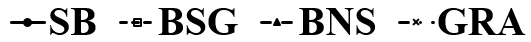  **Figure S3.** Significant effect of Treatment×Period interaction on starch intake (panel a), water-soluble carbohydrates (WSC) intake (panel b), and retained nitrogen/nitrogen intake (Retained N/NI, panel c) from steers fed the experimental diets during the chamber measurement periods of the animal trial. Significances were declared at *P* < 0.05. Significant differences within periods are indicated with different superscript letters according to Fisher's Least Significant Difference (LSD) test. Error bars represent standard error of means (SEM). |

| 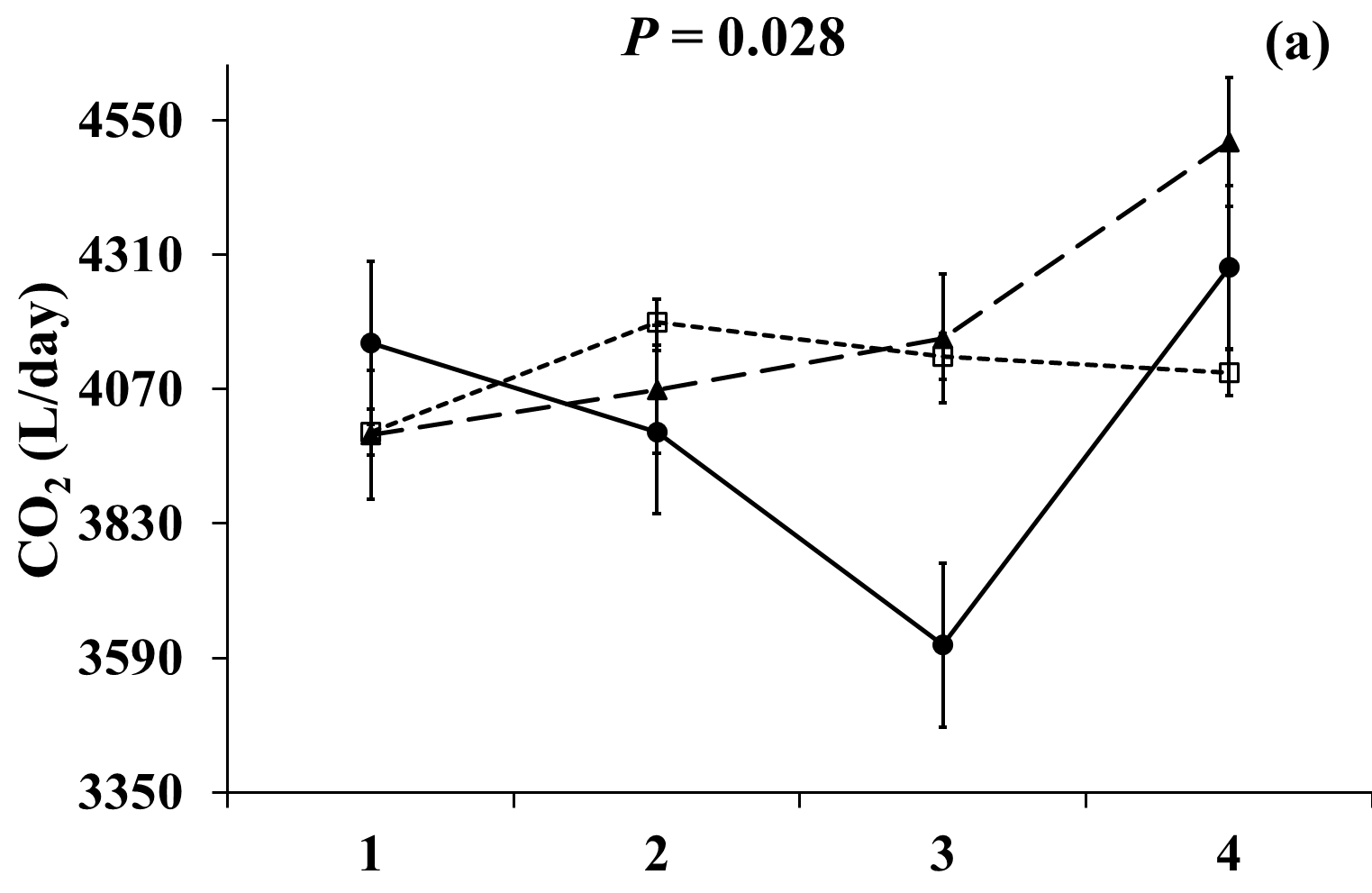 | 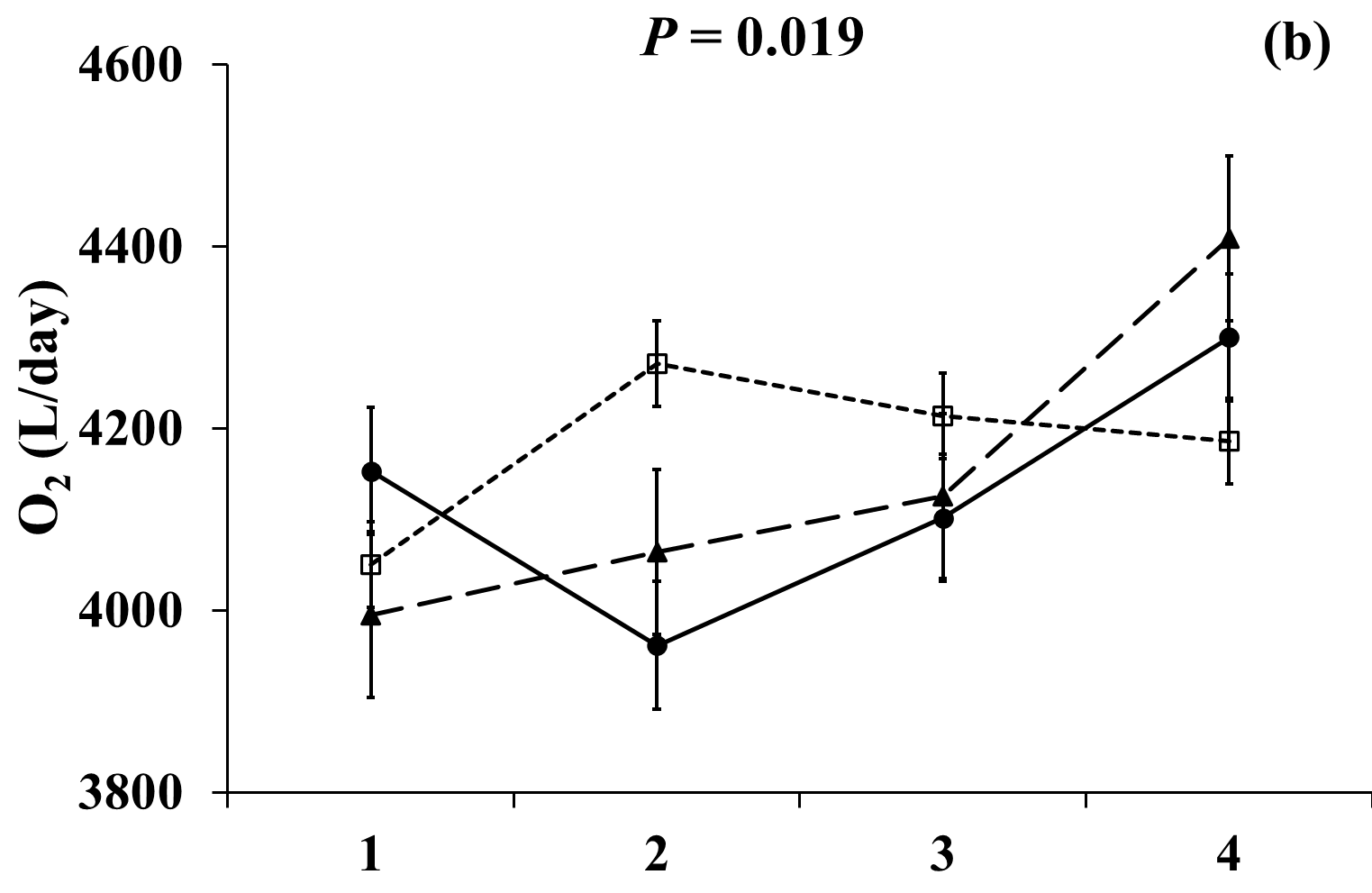 |
| --- | --- |
| 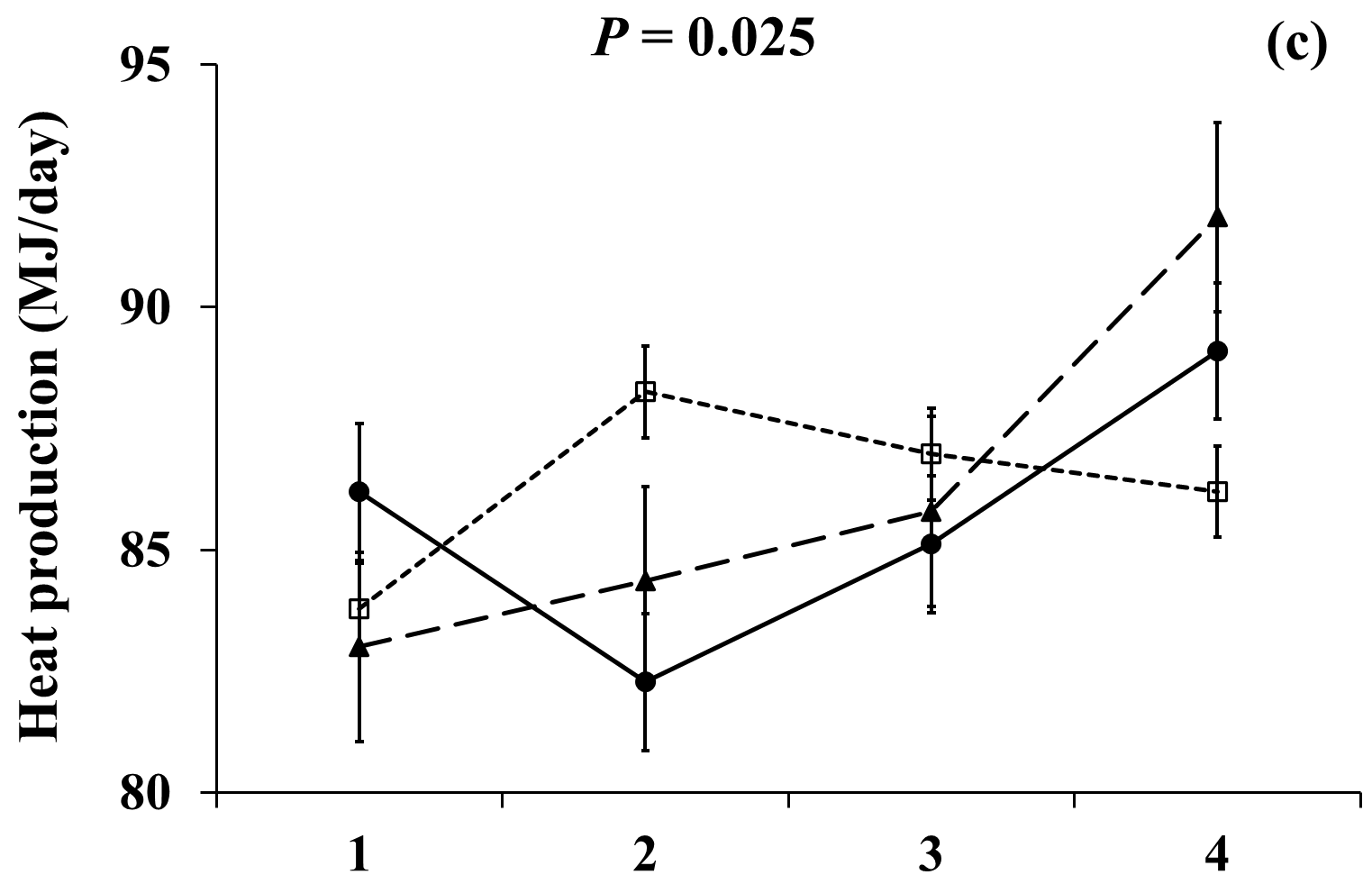 | 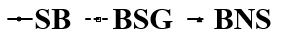  **Figure S4.** Significant effect of Treatment×Period interaction on CO_2_ (panel a), O_2_ (panel b), and heat production (panel c), from steers fed the experimental diets during the chamber measurement periods of the animal trial. Significances were declared at *P* < 0.05. Significant differences within periods are indicated with different superscript letters according to Fisher's Least Significant Difference (LSD) test. Error bars represent standard error of means (SEM). |

| 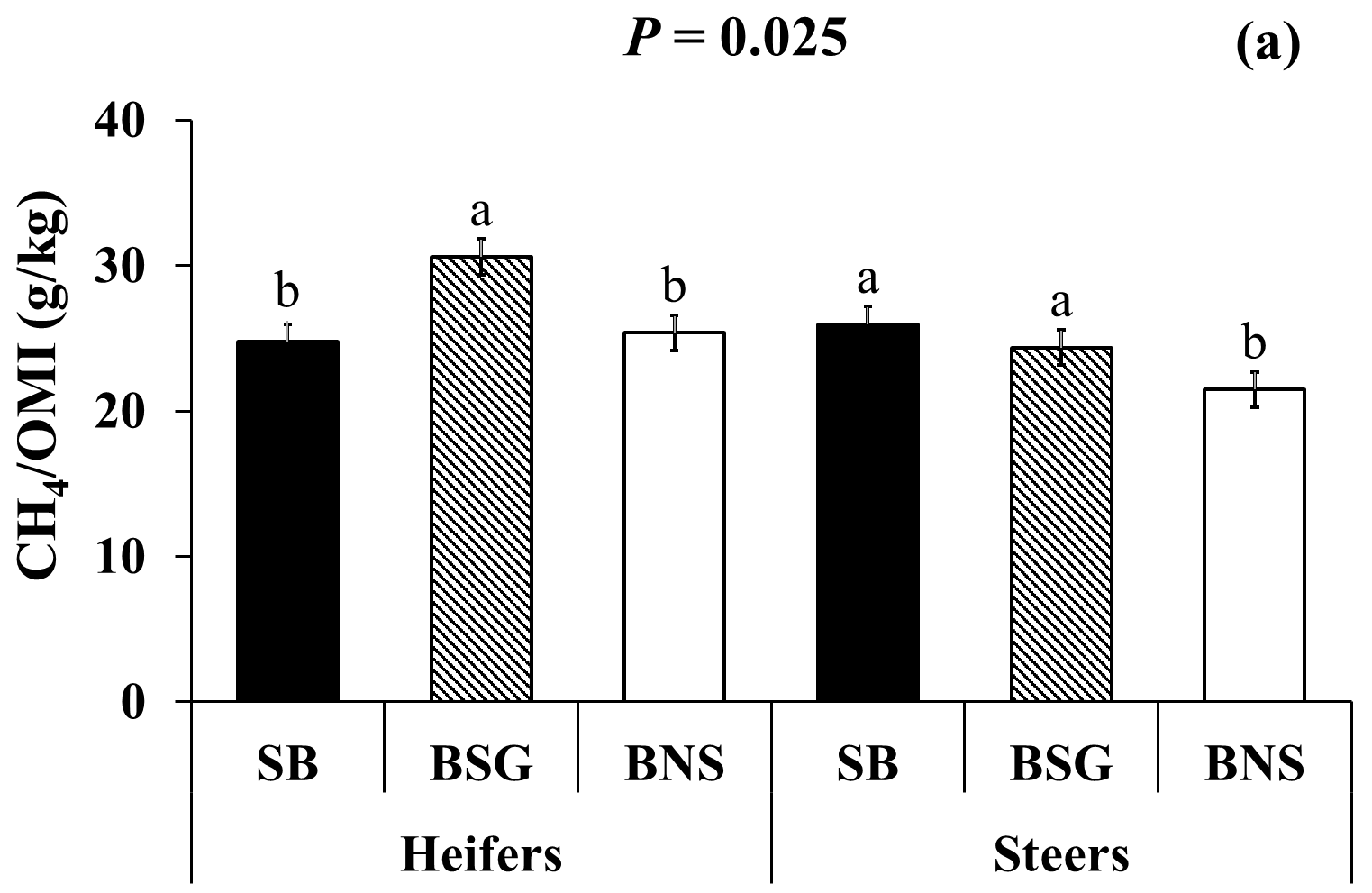 | 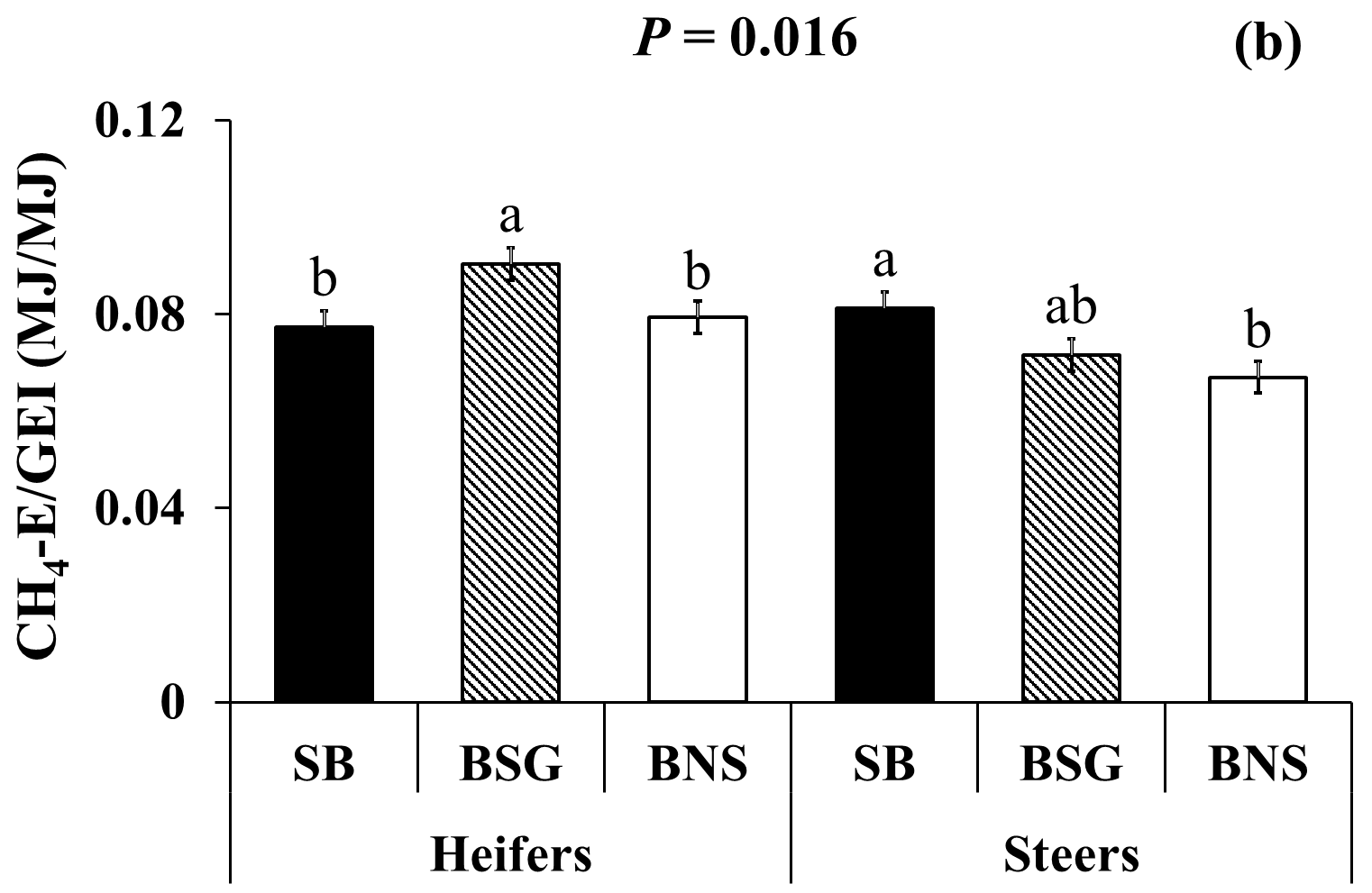 |
| --- | --- |
| 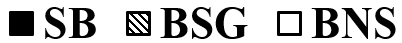 | |
| **Figure S5.** Significant effect of Treatment×Sex interaction on enteric CH_4_ emissions (expressed as g of CH_4_ per kg organic matter intake (OMI), panel a; and as MJ per MJ gross energy intake (GEI), panel b); in growing beef (heifers, steers) fed the experimental diets during the group-housed period of the animal trial. Significances were declared at *P* < 0.05. Significant differences within sex are indicated with different superscript letters according to Fisher's Least Significant Difference (LSD) test (*P* < 0.05). Error bars represent standard error of means (SEM). | |
